# Unique Metal–Ligand Proton Tautomerism Underlying the Reversible Electrocatalytic NAD^+^/NADH Interconversion

**DOI:** 10.1021/jacs.6c00789

**Published:** 2026-05-04

**Authors:** Gabriel Menendez Rodriguez, Leonardo Tensi, Elisa Boccalon, Cristiano Zuccaccia, Filippo De Angelis, Luca Rocchigiani, Alceo Macchioni

**Affiliations:** † Department of Chemistry, Biology and Biotechnology and CIRCC, 9309 University of Perugia, Perugia 06123, Italy; ‡ Department of Pharmaceutical Sciences, University of Perugia, Perugia 06123, Italy; § Department of Chemistry, Biology and Biotechnology and INSTM, University of Perugia, Perugia 06123, Italy

## Abstract

The NAD^+^/NADH electrochemical interconversion mediated by [Cp*Ir­(R–pyza)­Cl] (Cp* = pentamethylcyclopentadienyl, pyza = pyrazine-2-carboxyamidate, R = H, **1** and R = Me, **2**) represents a rare example of a process occurring at nearly zero overpotential, *i.e*., under conditions of reversible electrocatalysis. Here, we provide compelling evidence that the initial reduction of **1** and **2** is ligand-centered and leads to dearomatized and protonated complexes **1_LH** and **2_LH**, respectively, where the iridium center remains in its original oxidation state. Successively, **1_LH** and **2_LH** undergo a peculiar Metal–Ligand Proton Tautomerism (MLPT) leading to the metal hydride species **1_H** and **2_H**, respectively, without any change in the Ir oxidation state. Ultimately, **1_H** and **2_H** transfer a hydride to NAD^+^ producing NADH. Remarkably, **1_LH**/**2_LH** and **1_H**/**2_H** showed to be sufficiently stable to allow direct observation of their interconversion by NMR spectroscopy and quantification of accurate thermodynamic and kinetic parameters for such transformation. This work provides solid evidence for the beneficial role of such unique MLPT in enabling reversible electrocatalysis.

## Graphical abstract

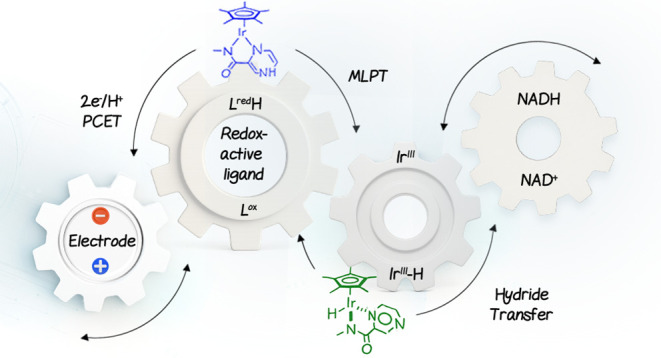

## Introduction

Metal–ligand proton tautomerism (MLPT), namely a proton shift between a metal center M and a ligand L in coordination complexes, is of fundamental importance in many (bio)­catalytic transformations. For instance, a series of intramolecular MLPT facilitate the formation of low-valent metal hydrides in hydrogenases through an initial protonation of the ligand.^1–6^ This process also inspired the design of many synthetic systems that incorporate proton relays exhibiting remarkable performance as catalysts for H_2_ evolution.^7–10^

From a fundamental point of view, typical MLPTs involve the decrease of the formal oxidation state and (a) coordination number of the metal by two units and reduction of the denticity (κ^m^) or hapticity (η^m^) of L by one (eq 1), or (b) the coordination number of the metal by one unit and shift of the positive charge onto a L ligand bearing a basic functionality (B) that can be protonated (eq 2).^6,11–19^ Examples of MLPT occurring according to (a) (eq 1), which can be likened to an intramolecular reductive elimination, have been reported by Miller^20^ and Blakemore^17^ for the transformation of [(η^5^–Cp*)­RhH­(bpy)] complexes (bpy = 2,2′–bipyridine) into [(η^4^–Cp*H)­Rh­(bpy)] and Goldberg^11,12,14^ for iridium complexes bearing the bifunctional 2–(di*tert*-butylphosphaneyl)–1H–imidazole ligand. On the other hand, a MLPT as illustrated in eq 2, path (b) has been suggested by Goldman for *para–*N–pyridyl-based PNP pincer iridium complexes^21^ and nicely demonstrated by Bullock for [Fe­(P^Et^N^Me^P^Et^)­(CO)_3_H]­X, which tautomerizes to [Fe­(P^Et^NH^Me^P^Et^)­(CO)_3_]­X,^13^ with the counterion X^–^ playing a key role.^22^

Hypothetically, an additional path (c), in which both the proton and the two electrons of the M–H bond are transferred into L^ox^–B and the metal center remains in its original oxidation state, can be proposed for MLPT. (eq 3). At first glance, eq 3 may appear analogous to the net intramolecular transfer of H^–^ typically invoked in hydride migratory insertion reactions.^23,24^ However, this process is mechanistically distinct from the MLPT pathway depicted in eq 3, involving the transfer of a proton (H^+^) from M–H bond to a basic site on the ligand, accompanied by intramolecular redistribution of the two electrons associated with the M–H bond.^25–30^ While this is potentially viable, it has never been observed to the best of our knowledge.
1

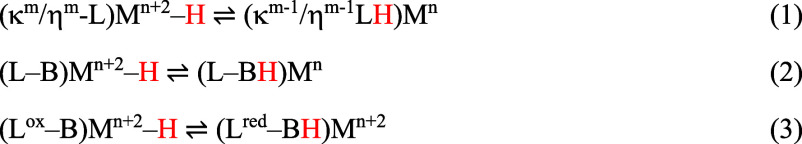




Herein, we report compelling evidence that hydride species (**1_H**/**2_H**), generated from [Cp*Ir­(R–pyza)­Cl] complexes (Cp* = pentamethylcyclopentadienyl, pyza = pyrazine–2–carboxyamidate, R = H, **1** and R = Me, **2**), undergo MLPT through path (c), yielding the ligand-reduced species **1_LH**/**2_LH** (Scheme 1).

**Scheme 1:**
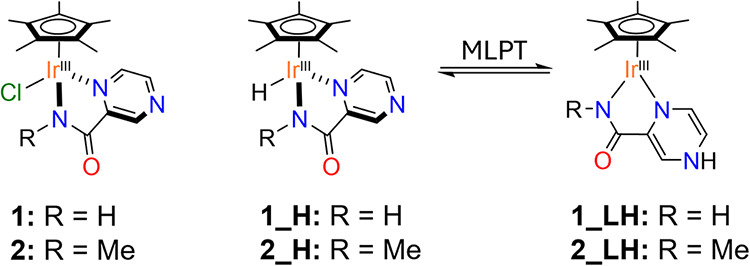
**Chemical Structure of 1/2, 1_H**/**2_H** and **1_LH/2_LH**

**1** is a member of a family of catalysts for NADH regeneration^31–36^ that we have developed over the last years through a mechanistically driven approach.^37–42^ It shows remarkable performance in both chemical regeneration of NADH, using phosphite as hydride donor,^41^ and electrochemical NADH regeneration.^39^ Furthermore, as reported in our previous work^39^, **1** acts as reversible electrocatalyst for NAD^+^/NADH interconversion, having the rather unique feature of catalyzing both NAD^+^ reduction and NADH oxidation with high selectivity and minimal overpotential. Electrocatalysts possessing such capability, *i.e*. that of facilitating a reaction in both the forward and reverse directions, in response to a small departure from equilibrium potential, are termed “reversible” and play a crucial role in energy storage, energy conversion, and sustainable chemistry, as they enable substantial reaction rates with minimal driving force, reducing overall energy consumption.^43–48^ In general, the design of a reversible electrocatalyst represents a significant challenge, as it is necessary that all steps of the catalytic cycle, including protonation, deprotonation, and electron transfer processes, proceed reversibly. Strikingly, only a handful of molecular systems have been reported so far exhibiting such a peculiar property.^49–58^

With the aim of elucidating the origin of the beneficial effect of the pyza ligand in NAD^+^/NADH interconversion, we conducted an in depth electrochemical and NMR study for both **1** and **2**. We discovered that the success of pyza stems from its ability to accept two electrons from the cathode and a proton from bulk solution and to deliver all of them (*i.e*. an equivalent of a hydride in terms of overall electron and proton count) to the metal center, leaving unaltered the oxidation state of the metal (eq 3). The latter process occurs through a reversible dearomatization/rearomatization of the pyza-ring, closely analogous to the behavior of the NAD^+^/NADH system (Scheme 2). Formally, the two electrons stored into the pyza ligand locate in the four carbon atoms of pyrazine formally decreasing their oxidation state from +0.5 to 0.

**Scheme 2:**
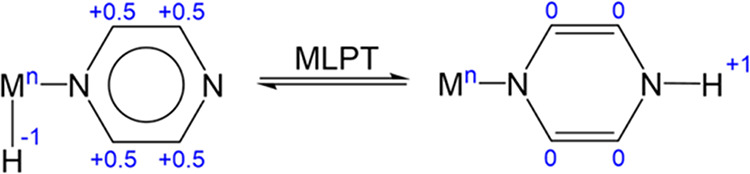
Formal Oxidation States Accompanying the Aromatization/De-Aromatization Process of the Pyza Ring during MLPT

Remarkably, both tautomeric forms are stable in buffered water for the previously reported catalyst **1**, as well as for **2**, a novel complex synthesized for the purpose of this work (Scheme 1). This allowed a complete NMR characterization of both isomers and the determination of thermodynamic and kinetic parameters of the MLPT process, along with clear indications of its reaction mechanism.

## Results and Discussion

### Synthesis and Characterization

Complex **1** was prepared as previously reported.^39^ Complex **2** was synthesized by reacting the dimeric iridium precursor, [Cp*IrCl_2_]_2_, with 2 equiv of the ligand in methanol, at room temperature for 4 h, in the presence of 2 equiv of KOH. The dark red solution was dried under vacuum and dichloromethane was added to the solid residue. The resulting suspension was filtered to remove KCl and the filtrate was layered with pentane to afford red crystals of **2**. Compound **2** was fully characterized in solution by 1D and 2D NMR spectroscopy and in the solid state by single crystal X–ray diffraction (Figure 1a).

**Figure 1:**
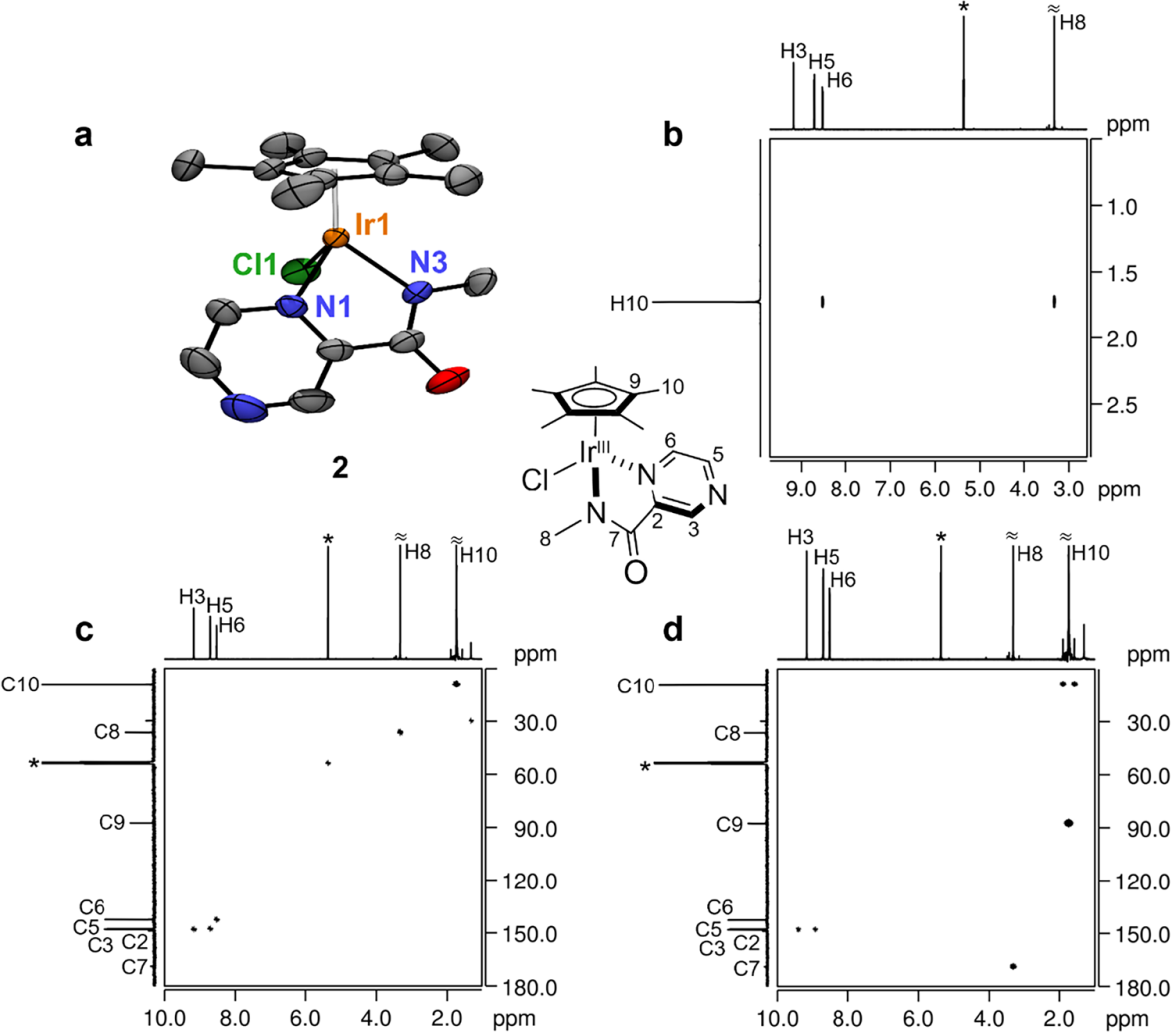
(a) ORTEP drawing of **2** (ellipsoids at 50% probability, solvent molecule and hydrogens omitted for clarity). Color Code: C = Gray, Ir = Orange, Cl = Green, *N* = Blue, O = Red. Relevant bond lengths and angles: Ir1–N1 = 2.088(2) Å, Ir1–N3 = 2.079(2) Å, Ir1–Cl1 = 2.4052(8) Å, Ir1–Cp_centroid_ = 1.788 Å, N3–Ir1–N1 = 76.53(9)°, N3–Ir1–Cp_centroid_ = 132.28°, N1–Ir1–Cp_centroid_ = 131.85°. (b) A section of the ^1^H NOESY NMR spectrum showing the NOE contacts between H6/H8 and H10 protons (CD_2_Cl_2_, 298 K). (c) ^1^H,^13^C HSQC spectrum of **2** showing the direct scalar correlations of aromatic protons and carbons (CD_2_Cl_2_, 298 K). (d) ^1^H,^13^C HMBC spectrum of **2** showing long-range scalar correlations between protons and carbons resonances.

The ^1^H NMR spectrum of **2** in CD_2_Cl_2_ displays a single set of five resonances, consistent with its molecular structure. The two singlets observed at δ_H_ = 3.47 and 1.66 ppm, integrating for 3 and 15 protons, respectively, were assigned to H8 and H10. The aromatic proton H6 (δ_H_ = 8.48 ppm) was identified due to its selective NOE with H10, further confirming the coordination of the pyza ligand to the Cp*Ir fragment (Figure 1b). Proton H5 was assigned through the observation of a strong dipolar interaction with H6 (not shown in Figure 1b), allowing the remaining aromatic signal to be attributed to H3. Assignment of the carbon resonances was achieved via scalar connectivity analysis using ^1^H,^13^C HSQC and ^1^H,^13^C HMBC NMR spectra (Figure 1c–d). Asterisk denotes the residual signal of the solvent.

**2** crystallized together with a molecule of water, likely deriving from the use of nonanhydrous solvents, with a monoclinic P2(1)/n unit cell. The complex features the classical piano stool geometry, with the N–Me–pyza acting as a bidentate ligand with both the nitrogen at position 1 and that of the carboxamide bound to the iridium. From the structural point of view, no significant changes in the bond lengths and angles were observed with respect to **1**,^39^ suggesting that the presence of the Me moiety is not relevantly affecting the electronic and steric environment of the metal center.

### Electrochemical Studies

The redox behavior of **2**, which in aqueous buffered solution is likely present as the aqua complex formed by displacement of the labile Cl^–^ ligand, was examined by cyclic voltammetry. As shown in Figure 2a, the CV obtained in Britton–Robinson buffer (BRB, 0.04 M, pH 7)^59^ exhibits a single reversible wave with a half–wave potential (*E*_1/2_) of –0.275 V, which is 12 mV more positive than the corresponding redox wave of **1** (*E*_1/2_ = –0.287 V) under the same pH conditions.^39^ The process is diffusion-controlled, as indicated by the linear dependence of the peak reduction current on the square root of the scan rate (Figure 2b). To further study the electron transfer process, additional CVs at varying pH were recorded for **1** and **2** (Figure 2c,e). The resulting Pourbaix diagrams show linear decreases in redox potential with pH, with slopes of –30 and –32 mV/pH for **1** and **2**, respectively, consistent with a 2e^–^/1H^+^ proton-coupled electron transfer (PCET) process (Figure 2d,f).

**Figure 2:**
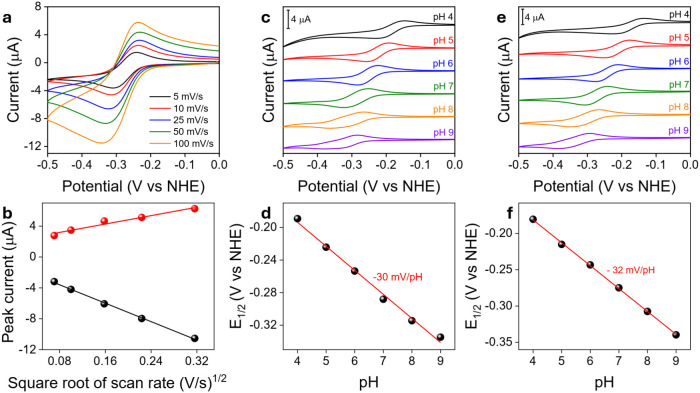
(a) Cyclic voltammograms of **2** recorded at pH 7 at different scan rates (*v*). (b) Plot of cathodic (black) and anodic (red) peak currents of **2** (red) as a function of the square root of the scan rate. (c, e) Cyclic voltammograms of **1** (c) and **2** (e) at different pH values recorded at 5 mV/s. (d, f) Corresponding Pourbaix diagrams of **1** (d) and **2** (f). Conditions: [Ir] = 0.5 mM, BRB (0.04 M), N_2_ atmosphere, 298 K.

The electrocatalytic activity of **2** to mediate the reversible interconversion of NAD^+^ and NADH was then investigated by varying pH from 6 to 8 and substrate concentration (Figure 3). When examining the effect of pH on the reduction of NAD^+^, a clear decreasing trend in catalytic current was observed with increasing pH, indicating reduced efficiency under more basic conditions. Specifically, at pH 6, the reduction peak current was approximately –9.7 μA, while at pH 7 it dropped to –7.6 μA, and further decreased to –4.7 μA at pH 8. In contrast, the oxidation of NADH showed only a slight increase in current across the same pH range, rising modestly from +3.2 μA at pH 6 up to +4.5 μA at pH 8, suggesting that NADH oxidation is relatively less sensitive to changes in pH. The effect of substrate concentration on the electrocatalytic activity of **2** was studied at pH 7. Initially, as substrate concentration increased, both reduction and oxidation currents rose, reflecting enhanced electrocatalytic activity. For example, when the NAD^+^ concentration increased from 1 mM to 2 mM, the reduction current passed from –5.8 to –6.8 μA. However, further enhancement in concentration to 3 mM resulted in no substantial change in current, with the reduction current plateauing around –7.1 μA. A similar trend was observed for the oxidation current, which rose from +4.2 μA at 1.0 mM to +4.9 μA at 2.0 mM and then remained nearly constant upon further increasing NADH concentration. These results suggest that beyond a certain substrate concentration threshold, the hydride transfer to NAD^+^ (or from NADH) is no longer the rate-limiting step in the catalytic process. Furthermore, the observed decrease in oxidation current upon enhancing the NAD^+^ concentration (Figure 3d) suggests that excess of NAD^+^ might hinder the catalytic turnover of NADH oxidation, pointing to inhibitory interactions that adversely impact the overall reaction kinetics. Further experiments were conducted in the presence of equimolar amounts of NAD^+^ and NADH (1.5 mM each). As shown in Figure 3f, the resulting current response displays the sigmoidal shape typical of reversible catalytic systems,^43^ with the current becoming independent of potential at both extremes. Under these conditions, the observed *plateau* current of NAD^+^ reduction was *i*_p_^red^ = –6.0 μA, whereas that of NADH oxidation was *i*_p_^ox^ = 2.2 μA. The ratio of these *plateau* currents (|*i*_p_^red^/ *i*_p_^ox^| = 2.8), referred to as the “catalytic bias” or “catalytic preference”,^60–62^ indicates that **2** is significantly more active in NAD^+^ reduction than in NADH oxidation, as previously observed for complex **1**.^39^

**Figure 3:**
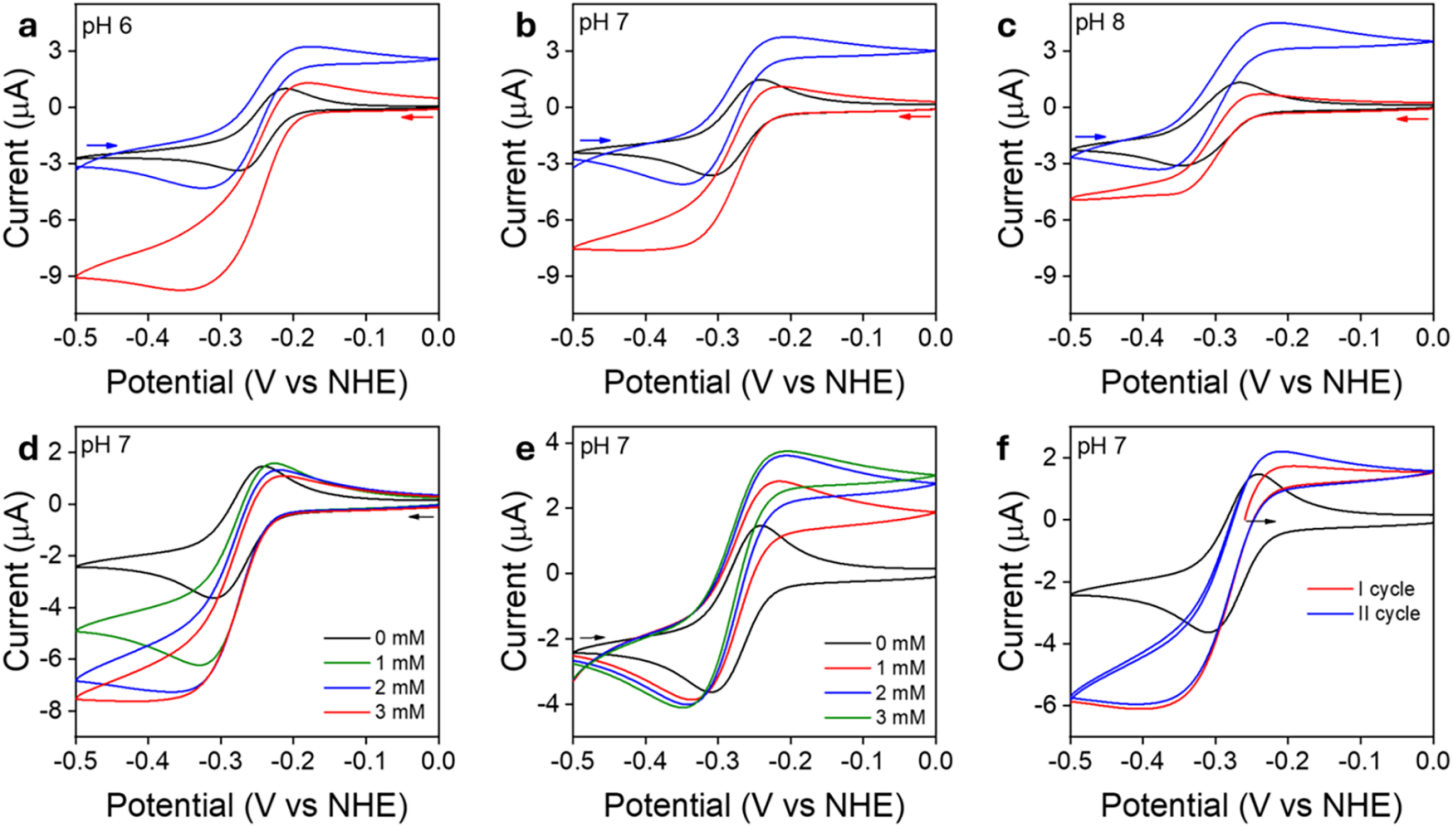
(a, b, c) CVs of **2** (0.5 mM) at different pH values in the absence (black) and presence of 3.0 mM of NAD^+^ (red) or NADH (blue). (d, e) CVs of **2** (0.5 mM) in the presence of different concentrations of NAD^+^ (d) and NADH (e). (f) CVs of **2** (0.5 mM) in the presence of both NAD^+^ (1.5 mM) and NADH (1.5 mM) starting from the open circuit potential (–0.26 V vs NHE). Black trace corresponds to the CV of **2** (0.5 mM) in the absence of substrate. Arrows indicate the scanning direction. All scans were recorded under N_2_ atmosphere in BRB (0.04 M) at a scan rate of 5 mV s^–1^.

### NMR Characterization of Reduced Intermediates

The reduction of Ir–pyrazinamidate complexes was investigated by multinuclear and multidimensional NMR spectroscopy, employing potassium formate as the reducing agent. NMR studies focused mainly on complex **2**, which features a N–Me group rather than an N–H moiety, thus offering an additional diagnostic signal for spectroscopic characterization, as well as for its greater stability under the experimental conditions *(vide infra)*, which enabled more reliable monitoring of the reduced species. The ^1^H NMR spectrum of a solution of **2** (4 mM in 0.1 M PBS pH 7, H_2_O/D_2_O 90:10) and ca. 20 equiv of HCOOK, recorded at 298 K, showed two sets of resonances corresponding to two distinct species in a relative ratio of 80:20. The major of those exhibited a typical hydride resonance at –12.08 ppm (Ir–H) that dipolarly correlates with H10 (Figure 4a).^82,83^ All NMR evidence supports the assignment of the latter to the neutral hydride complex [Cp*Ir­(Me–pyza)­H] (**2_H**). Interestingly, the least abundant species displays a significant shift of the resonances in comparison with those of both **2** and **2_H**. For instance, pyrazine ring protons show signals in the range of 7.2 to 5.6 ppm (blue labeled H3, H5 and H6) instead of 8–8.5 ppm in **2_H** (green labeled). The ^1^H,^13^C-HSQC spectrum (Figure 4b) showed that the aforementioned protons are scalarly coupled with carbon resonances at δ_C_ = 136.8 ppm (C6), δ_C_ = 129.6 ppm (C3) and δ_C_ = 115.4 ppm (C3), which are clearly not consistent with an aromatic pyrazine ring (e.g., C3, C5 and C6 of complexes **2** and **2_H** resonate at δ_C_ = 142–148 ppm). Those observations strongly indicate that 2e^–^/1H^+^ reduction of the pyrazine ligand occurred, resulting in the formation of complex **2_LH** depicted in Figure 4.^63^ Further confirmation of this assignment was obtained by repeating the reaction of **2** with potassium formate in DMSO-*d*_*6*_, where N–H and O–H protons are usually observable. In this solvent, an additional resonance at 9.5 ppm was present in the ^1^H NMR spectrum, which was assigned to H4 based on its scalar coupling with H3 and H5, as observed in the ^1^H COSY spectrum (Figure S5d). Additional insights into the electronic changes accompanying pyrazine reduction were obtained from ^15^N NMR studies (Figure 4c and Supporting Information (SI)). In complexes **2** and **2_H**, the pyrazine nitrogen atoms N1 and N4 resonate at 244.9 and 337.7 ppm and 237.2 and 324.8 ppm, respectively, values consistent with a fully aromatic pyrazine ring.^64^ By contrast, in **2_LH**, both nitrogen resonances shift dramatically at lower frequency down to 171.4 (N1) and 105.5 ppm (N4), indicating a substantial increase in electron density at the pyrazine nitrogen atoms and loss of aromaticity (Figure 4c). Comparable behavior has been documented for pyridine ring nitrogen of NAD^+^ and related compounds.^65^ It has been demonstrated that the ^15^N chemical shift of the pyridine N1 position is highly sensitive to the redox state of the ring, exhibiting low frequency changes of over 100 ppm upon reduction of NAD^+^ to NADH. This close analogy, together with the changes observed in the ^13^C and ^1^H chemical shifts, collectively support the conclusion that the newly observed species corresponds to a reduced, protonated pyrazine complex, **2_LH**. Notably, the negative cross–peaks observed in the 2D–EXSY spectrum between the proton resonances of **2_H** and **2_LH** provide clear evidence for a dynamic equilibrium between these two species (Figure 4d). This observation strongly supports the occurrence of a Metal–Ligand Proton Tautomerism (MLPT) through path (c) as defined in the Introduction (eq 3). Similar results were obtained for **1** with formate. With the latter, the corresponding **1_H** and **1_LH** species were detected in lower yields, as the majority of the Ir centers (70%) accumulate in the form of a Cp* complex with an over–reduced pyrazine–amidate ligand (Figure S6). Although proton migration into the Cp* ligand might occur, as mentioned in the Introduction for Rh–complexes, no evidence of that was provided by NMR experiments.

**Figure 4:**
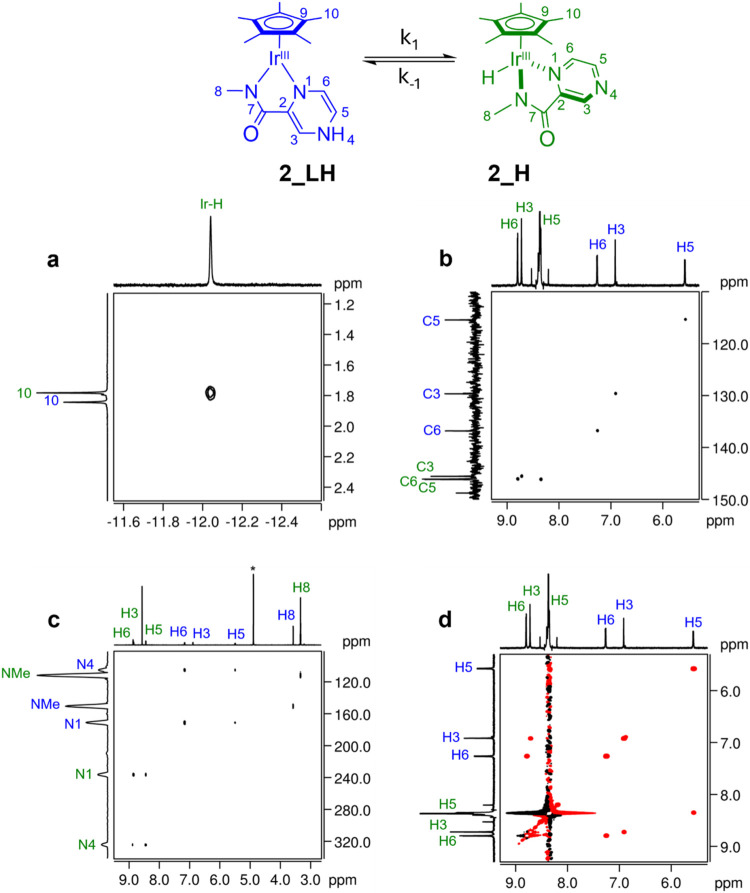
(a) A section of the ^1^H NOESY NMR spectrum showing the NOE contacts between the hydride resonance and H10 protons of **2_H**. (b) A section of the ^1^H,^13^C HSQC spectrum showing the direct scalar correlations of protons and carbons of the pyrazine ring. (c) A section of the ^1^H,^15^N HMBC spectrum showing scalar correlations between ^15^N and ^1^H nuclei of **2_H** and **2_LH**. (d) A section of the 2D EXSY NMR spectrum showing the exchange cross peaks between the resonances of **2_H** and **2_LH**. Conditions: 0.1 M PBS (pH 7), H_2_O/D_2_O 90:10, 298 K (a, b and d). (c) CD_3_OD, 298 K. Asterisk denotes the residual signal of the solvent.

### Thermodynamics and Kinetics of MLPT

Considering the peculiarity of this MLPT and its strict relationship with NAD^+^/NADH transformation, understanding the mechanism that governs **2_LH**/**2_H** interconversion is of great importance. Crucial information in this respect was obtained by analyzing the equilibrium between **2_LH** and **2_H** and the corresponding dynamic behavior in buffered water solutions at 278–298 K, where both complexes are stable. The presence of separated NMR resonances for both isomers not only allowed to experimentally determine the thermodynamic constant for the equilibrium depicted in Figure 4 (*K*_MLPT_), but also to measure the rate constants at equilibrium for the forward (*k*_1_) and backward (*k*_*–*1_) reactions, by quantifying the rate of magnetization transfer by 1D EXSY NMR spectroscopy.^66^ Experimental data are collected in Table 1.

**Table 1:** Thermodynamic and Kinetic Data Relative to the MLPT Depicted in Figure 4

entry	*T* (K)	*K*_MLPT_	*k*_1_ (s^–1^)	*k*_–1_ (s^–1^)
1^a^	278	2.6 ± 0.1	0.49 ± 0.05	0.19 ± 0.02
2^a^	283	2.9 ± 0.1	0.75 ± 0.07	0.25 ± 0.02
3^a^	288	3.3 ± 0.2	0.98 ± 0.09	0.27 ± 0.02
4^a^	293	3.7 ± 0.2	1.3 ± 0.1	0.33 ± 0.03
5^a^	298	4.0 ± 0.2	1.7 ± 0.2	0.39 ± 0.03
6^b^	298	4.0 ± 0.2	1.3 ± 0.1	0.27 ± 0.02
		Δ*H*° = 3.6 ± 0.6	Δ*H* ^‡^ = 9.4 ± 0.6	Δ*H* ^‡^ = 5.1 ± 0.5
		Δ*S*° = 15 ± 2	Δ*S* ^‡^ = −26 ± 2	Δ*S* ^‡^ = −43 ± 2
		Δ*G*°_(298)_ = −1 ± 1	Δ*G*_(298)_^‡^ = 17 ± 1	Δ*G*_(298)_^‡^ = 18 ± 1

a Experiment performed in 0.1 M PBS (pH 7, H_2_O/D_2_O 90:10).

b Experiment performed in 0.1 M Tris/HCl buffer (pH 7, H_2_O/D_2_O 90:10). Δ*H* and Δ*G* are expressed in kcal mol^–1^, whereas Δ*S* in cal K^–1^ mol^–1^.

The observed trend in equilibrium constants reveals a clear dependence on temperature. Specifically, the value of *K*_*MLPT*_ increases from 2.6 ± 0.1 at 278 K to 4.0 ± 0.2 at 298 K (Table 1, entries 1–4), consistently with the behavior expected for an endothermic reaction. Δ*H*° and Δ*S*° values were derived from the van’t Hoff plot of ln*K*_MLPT_ vs 1/*T* (Figure 5a) and found to be 3.6 ± 0.6 kcal mol^–1^ and 15 ± 2 cal mol^–1^ K^–1^, respectively. The positive entropy change might be associated with a decrease in water organization in the second coordination sphere, tentatively attributed to stronger hydrogen bonding interactions between the NH moiety of **2_LH** and water, relative to those involving the unprotonated pyrazine ring of **2_H**. The standard Gibbs free energy at 298 K (Δ*G*°_(298)_), calculated using the relation Δ*G*° = Δ*H*° – *T*Δ*S*°, was –1 ± 1 kcal mol^–1^. This value is in excellent agreement with the one obtained independently from the equilibrium constant via Δ*G*° = –RTln­(*K*_MLPT_), which yielded –0.82 kcal mol^–1^. The consistency between these values further validates the reliability of the van’t Hoff analysis and the thermodynamic interpretation of the equilibrium data.

**Figure 5:**
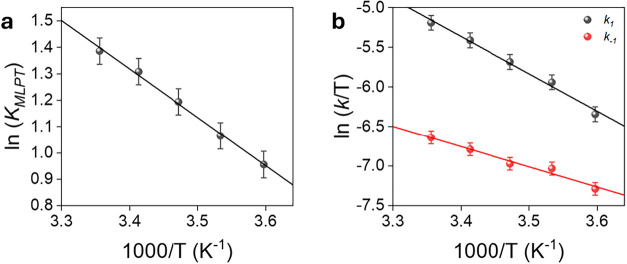
(a) Van’t Hoff plot of ln*K*_MLPT_ vs 1/T and (b) Eyring plots ln­(*k*/T) vs 1/T for *k*_1_ (black) and *k*_–1_ (red).

As far as the rate constants at equilibrium are concerned, the forward (*k*_1_) rate constant was observed to increase significantly with temperature, ranging from 0.49 ± 0.05 to 1.7 ± 0.2 s^–1^ over the 278–298 K interval. In contrast, the reverse rate constant (*k*_*–*1_) exhibited a more modest variation, ranging from 0.19 ± 0.02 to 0.39 ± 0.03 s^–1^ across the same temperature range. Activation parameters for both the forward and reverse reactions were determined by Eyring plot analysis of the kinetic data (Figure 5b). For the forward reaction, the activation enthalpy (Δ*H* ^‡^) and activation entropy (Δ*S* ^‡^) were found to be 9.4 ± 0.6 kcal mol^–1^ and –26 ± 2 cal K^–1^ mol^–1^, respectively, resulting in an activation Gibbs free energy (Δ*G*_(298)_^‡^) of 17 ± 1 kcal mol^–1^. The corresponding values for the reverse reaction were Δ*H* ^‡^ = 5.1 ± 0.5 kcal mol^–1^, Δ*S* ^‡^ = –43 ± 2 cal K^–1^ mol^–1^ and Δ*G*_(298)_^‡^ = 18 ± 1 kcal mol^–1^. The higher Δ*H* ^‡^ for the forward reaction reflects a greater energetic barrier, consistent with the observed stronger temperature dependence of *k*_1_ compared to *k*_*–*1_. The negative values of Δ*S* ^*‡*^ for both directions indicate that the transition state is more ordered than both **2_LH** and **2_H**. This substantial entropic penalty is characteristic of an associative process involving a highly organized transition structure, likely arising from the formation of an extensive hydrogen–bonding network between the reacting species and components of the solvent and/or buffer system. Overall, these activation parameters, together with the equilibrium thermodynamics, point to a moderately endothermic and entropically disfavored transition state.

To further probe the role of hydrogen bonding interactions and the influence of buffer composition, the kinetics of the MLPT reaction were investigated in an alternative buffer system, namely Tris/HCl buffer (0.1 M, pH 7). Under these conditions, the equilibrium constant remained effectively unchanged, indicating that the thermodynamic position of the reaction is not buffer–dependent. However, both the forward and reverse rate constants were lower than those measured in phosphate buffer under identical conditions (Table 1, entry 6). This observation highlights the active participation of the buffer components in facilitating the reaction kinetics. The reduced rate constants in Tris/HCl might suggest that phosphate ions serve as more effective hydrogen bond donors and/or acceptors, thereby stabilizing the transition state and lowering the activation barrier.^67,68^ In addition, formate ions were also found to facilitate the tautomerization process, as evidenced by a clear concentration-dependent increase in both the forward (*k*_1_) and reverse (*k*_*–1*_) rate constants (Figure S7). These results provide further evidence that specific hydrogen bonding interactions involving buffer and/or cosolute species can significantly modulate the kinetics of MLPT, leaving the overall thermodynamic equilibrium unchanged.

### Hydride Transfer Reaction

Further experiments were performed to investigate the thermodynamic and kinetic properties of the hydride transfer reaction between **2** and NADH (eq 4):

4 $$2\_H+{\mathrm{NAD}}^{+}⇌2+\mathrm{NADH}$$

The equilibrium constant (*K*_HT_) of the above reaction was determined at 298 K by ^1^H NMR spectroscopy. Equimolar amounts of **2** and NADH were reacted in BRB buffer (0.04 M, pH 7), the same buffer system employed in all electrochemical experiments. The relative intensities of the ^1^H NMR resonances, together with the known reaction stoichiometry, were used to calculate the molar fraction of each species. These values were then applied to eq 5 to determine the equilibrium constant *K*_HT_ (see SI).

5 $$K_{HT}=\frac{k_{2}}{k_{-2}}=\frac{\left[2\right]\left[\mathrm{NADH}\right]}{[2\_H]\left[{\mathrm{NAD}}^{+}\right]}$$

Under these conditions, a *K*_HT_ value of 0.3 ± 0.1 was obtained, corresponding to a Δ*G*°_HT(298)_ of 0.7 ± 0.2 kcal mol^–1^, indicating that the equilibrium is slightly shifted toward the reactants. This result contrasts significantly with that reported previously for complex **1**, for which a *K*_HT_ = 3 and a Δ*G*°_HT(298)_ = –0.6 kcal mol^–1^ were obtained. The discrepancy is attributed to the different buffer system used in the earlier study (Tris/HCl 0.1 M, pH 7). In that case, the higher equilibrium constant results from substitution of the coordinated water ligand in compound **2** by Cl^–^ anions upon dissolution, which provides additional stabilization of products, thereby shifting the equilibrium toward product formation. Indeed, when the same experiment was repeated with **2** under the same conditions used in our previous study (Tris/HCl 0.1 M, pH 7), a *K*_HT_ value of 3 ± 1 was obtained, identical to that observed for **1**. This result indicates that replacing the NH proton with a methyl group does not significantly affect the thermodynamics of the hydride transfer process. Therefore, the *K*_HT_ and Δ*G*° values reported here should be considered as effective values, specific to the current experimental conditions. The same caveat applies to the hydricity of **2_H**, calculated to be Δ*G*°_H–_ = 29.6 ± 0.2 kcal mol^–1^ in BRB (0.04 M, pH 7, see SI), which is slightly higher than that of NADH (28.9 kcal mol^–1^).^69–71^

Initial attempts to investigate the kinetics of the reaction between **2** and NADH were conducted using time–resolved UV–vis spectroscopy. A solution of **2** (0.1 mM) in BRB buffer (0.04 M, pH 7) was treated with 20 equiv of NADH, and spectra were recorded every 0.5 s, which corresponds to the minimum acquisition interval permitted by the spectrophotometer. In the 200–400 nm range, no spectral changes could be resolved due to the intense absorbance of NADH at this high concentration (2.0 mM). However, upon NADH addition, a broad absorption band immediately appeared in the visible region (centered around 480 nm), indicating the rapid, essentially instantaneous (within <0.5 s), formation of a new species (Figure 6a). Over the next 100 s, two distinct absorption bands at 446 and 537 nm gradually emerged and increased in intensity, after which no further spectral changes were observed, suggesting the formation of a stable reaction mixture. The almost identical absorption features observed after reacting **2** with 20 equiv of potassium formate, confirm that this final mixture corresponds to the **2_H**/**2_LH** equilibrium (Figure S8). Notably, the time–dependent spectral evolution is consistent with a two–step process (Figure 6b): fast hydride transfer from NADH to **2** forming **2_H**, followed by a slower metal–ligand proton tautomerization yielding **2_H**/**2_LH** equilibrium mixture. However, due to the limited time resolution of the spectrometer, kinetic parameters for the initial hydride transfer could not be determined under these conditions.

**Figure 6:**
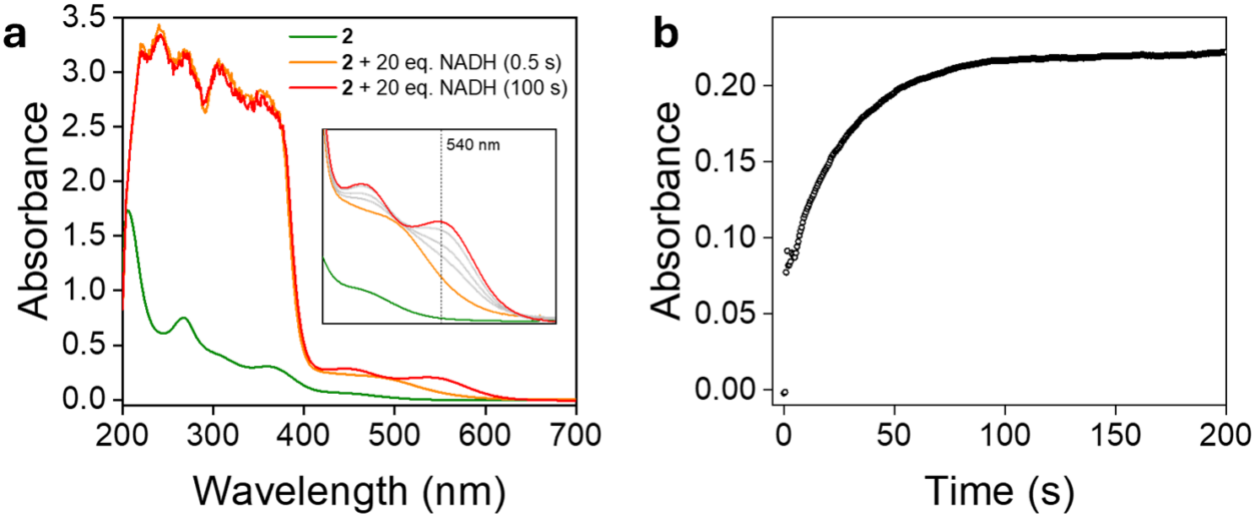
(a) Evolution of the UV–vis spectrum in the reaction of **2** (0.1 mM) with 20 equiv of NADH in BRB (0.04 M, pH 7) and (b) corresponding plot of the absorbance at 540 nm versus time.

The hydride transfer rate constants for the forward (*k*_2_) and reverse (*k*_*–*2_) reactions were determined using dynamic NMR spectroscopy under equilibrium conditions, following an analogous approach to that used for investigating the kinetics of the MLPT process. In this case, a solution containing NAD^+^ (3.0 mM), NADH (3.0 mM) and **2** (0.5 mM) in BRR (0.04 M, pH 7) was prepared, and the magnetization transfer rates between NAD^+^ and NADH were measured by monitoring the chemical exchange between magnetically distinct resonances of the two species. The second order rate constant of the forward (*k*_2_) and backward (*k*_–2_) reactions (eq 2), were then derived by dividing the forward and backward magnetization transfer rates by the concentrations of **2_H** and **2**, respectively. The resulting values, *k*_2_ = 1400 ± 200 s^–1^ M^–1^ and *k*_–*2*_ = 4300 ± 600 s^–1^ M^–1^, yield an equilibrium constant (*K*_HT_ = *k*_2_/*k*_*–*2_) of 0.33, in excellent agreement with the value of 0.3 determined from integration of the resonances in the ^1^H NMR spectrum. Notably, the fact that *k*_–2_[NADH] (8.6 s^–1^ at [NADH] = 2 mM) ≫ *k*_–1_ (0.39 s^–1^) supports the kinetic model inferred from UV–vis spectroscopy, in which the reaction of **2** with 20 equiv of NADH leads to fast **2_H** accumulation, followed by a slower tautomerization to **2_LH**.

### Metal or Ligand Centered Redox Process

Although the stoichiometry of reduction of **2** is well–defined (2e^–^, 1H^+^ PCET) and ultimately leads to a mixture of **2_LH** and **2_H**, the precise locus of the redox event, whether centered on the metal or ligand, remains ambiguous. This uncertainty is common in systems featuring redox–active ligands and metal centers with different accessible oxidation states.^72,73^ In the present case, reduction of the Ir­(III) center to Ir­(I) is likely feasible within the applied potential window. Moreover, it is well established that pyrazine^74^ and its derivatives^75,76^ exhibit redox activity, even when coordinated to metal ions,^84–77^ thus complicating the assignment of the redox event to a specific molecular fragment.

Two limiting mechanistic scenarios can thus be postulated: (A) metal–centered reduction and protonation to yield **2_H**, which subsequently undergoes tautomerization to generate **2_LH** (eq 6); or (B) ligand–centered process leading initially to **2_LH**, followed by tautomerization to form **2_H** (eq 7).

$$2+2e^{-}+H^{+}⇌2\_H⇌2\_\mathrm{LH}\;\left(\mathrm{scenario A}\right)$$ 6

7 $$2+2e^{-}+H^{+}⇌2\_\mathrm{LH}⇌2\_H\;\left(\mathrm{scenario B}\right)$$

Although both routes ultimately converge to a thermodynamic mixture of the two tautomers through metal–ligand proton tautomerism, discriminating between these pathways is crucial from a mechanistic perspective. If scenario A is correct, it implies that MLPT leading to **2_LH** constitutes a detrimental off–cycle process in NAD^+^/NADH interconversion, effectively subtracting catalytically active hydride species **2_H**. Conversely, if scenario B is operative, it would suggest that ligand–centered redox chemistry plays a direct and productive role in the catalytic mechanism, highlighting a rare and valuable example of electrocatalysis mediated by a multielectron redox–active ligand.^72,78,79^

These two mechanistic possibilities were discriminated by open circuit potentiometry (OCP) experiments.^80,81^ When a redox couple is present in solution, the OCP equals the Nernst potential of that couple and therefore directly reflects the thermodynamic ratio between the reduced and oxidized species, provided that equilibrium is established and electron transfer is fast. Thus, since the identity of the redox couple differs in the two scenarios (**2**/**2_H** for scenario A and **2**/**2_LH** for scenario B), the expression for the OCP necessarily differs as well. Specifically, (eq 8 and 9) are obtained, in which the pH term is already contained in the experimental value of *E*_1/2_.

8 $$\mathrm{OCP}=E_{1/2}-\frac{\mathrm{RT}}{\mathrm{nF}}\ln\frac{\left[2\_H\right]}{\left[2\right]},\left(\mathrm{scenario A}\right)$$

9 $$\mathrm{OCP}=E_{1/2}-\frac{\mathrm{RT}}{\mathrm{nF}}\ln\frac{\left[2\_\mathrm{LH}\right]}{\left[2\right]},\left(\mathrm{scenario B}\right)$$

These relationships can also be interpreted in reverse: if the ratio [**2_H**]/[**2**] or [**2_LH**]/[**2**] is known under equilibrium conditions, the corresponding OCP can be calculated. Since the equilibrium speciation of the system (*i.e*. the concentration of **2**, **2_H**, **2_H**, NAD^+^ and NADH) generated by the reaction of NADH with **2** can be determined for any given initial concentrations of both species (see Supporting Information), it is therefore possible to predict how the OCP should vary in each mechanistic scenario as a function of the equivalents of NADH reacted with **2**. These theoretical predictions, shown as blue and red traces in Figure 7a for scenarios A and B, respectively, enable mechanistic discrimination between the two pathways by directly comparing the calculated OCP values with those experimentally measured upon reacting NADH with **2**. In other words, the OCP analysis of the system under equilibrium can distinguish whether the major (**2_H**) or minor (**2_LH**) species is in the redox equilibrium with **2**.

**Figure 7:**
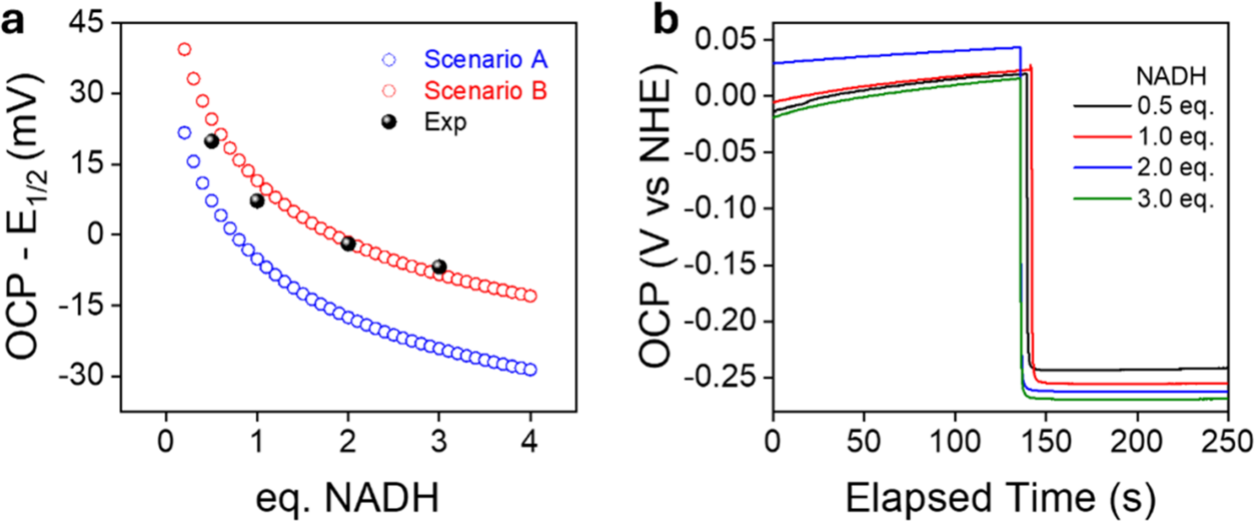
(a) Theoretical predictions of OCP variation as a function of NADH equivalents added to a solution of **2** (0.5 mM) in BRB (0.04 M, pH 7) for both mechanistic scenarios A (blue) and B (red), and experimental data (black). (b) OCP experiments for the reaction of **2** (0.5 mM) in BRB (0.04 M, pH 7) with different amounts of NADH.

In a typical OCP experiment, the electrochemical cell was filled with a solution of **2** (0.5 mM) in BRB buffer (0.04 M, pH 7), which was purged with nitrogen to remove dissolved oxygen and allowed to equilibrate under continuous stirring. The OCP was monitored for at least 120 s before NADH (0.5–4 equiv) was added. As shown in Figure 7b, each NADH addition resulted in an immediate drop in the OCP, which then gradually stabilized, reflecting the same trend observed in UV–vis spectroscopic studies. To ensure that equilibrium was reached, OCP values were taken in the *plateau* region, specifically 100 s after each NADH addition. Notably, the experiment showed excellent reproducibility across multiple independent replicates, underscoring the robustness of the methodology and the reliability of the observed electrochemical behavior (Figure S9). The measured potentials were then compared with the theoretical values predicted for each mechanistic scenario, based on Nernst equations incorporating the independently determined equilibrium constants *K*_HT_ and *K*_MLPT_. The excellent agreement between the experimental OCP data and the theoretical curve corresponding to scenario B (Figure 7a), not only provides compelling evidence that the redox process in this system is ligand–centered, but also independently validates the thermodynamic parameters derived from NMR spectroscopic studies.

### Proposed Mechanism

The electrochemical, spectroscopic, and potentiometric data collectively support the mechanism illustrated in Figure 8 for NAD^+^/NADH reversible interconversion. The process is initiated by a two–electron, one–proton reduction of **2_H**_**2**_ **O** (the most likely redox active form of catalyst in BRB), in which the redox event is centered on the ligand framework rather than the metal center, yielding **2_LH**, the protonated, ligand–reduced species. In the second step, **2_LH** undergoes an MLPT to form the hydride complex **2_H**. As shown by dynamic NMR experiments, the rate of this tautomerization is highly sensitive to buffer nature and cosolute concentration, underscoring the entropically disfavored nature of the transformation. Furthermore, cyclic voltammetry data reveal that increasing concentrations of NAD^+^ lead to suppression of the catalytic current associated with NADH oxidation. This observation suggests that the presence of substrate inhibits the MLPT step, likely by interfering with the establishment of essential hydrogen–bonding interactions between the catalytic species and the buffer or solvent components. Such interactions, potentially mediated by second–sphere coordination, appear to be crucial for enabling efficient proton transfer during the tautomerization step. The final step involves hydride transfer from **2_H** to NAD^+^, regenerating complex **2** and producing NADH. As evidenced by electrochemical and spectroscopic studies, this step is kinetically fast with respect to MLPT, implicating that the latter is the rate–determining step of the process. This assignment is fully consistent with the higher activity of **2** in the reduction of NAD^+^ relative to the oxidation of NADH, since the MLPT equilibrium (*K*_MLPT_ = [**2_H**]/[**2_LH**] = 4) favors the generation of the Ir–H species, which subsequently delivers hydride to NAD^+^, thereby promoting the reduction pathway.

**Figure 8:**
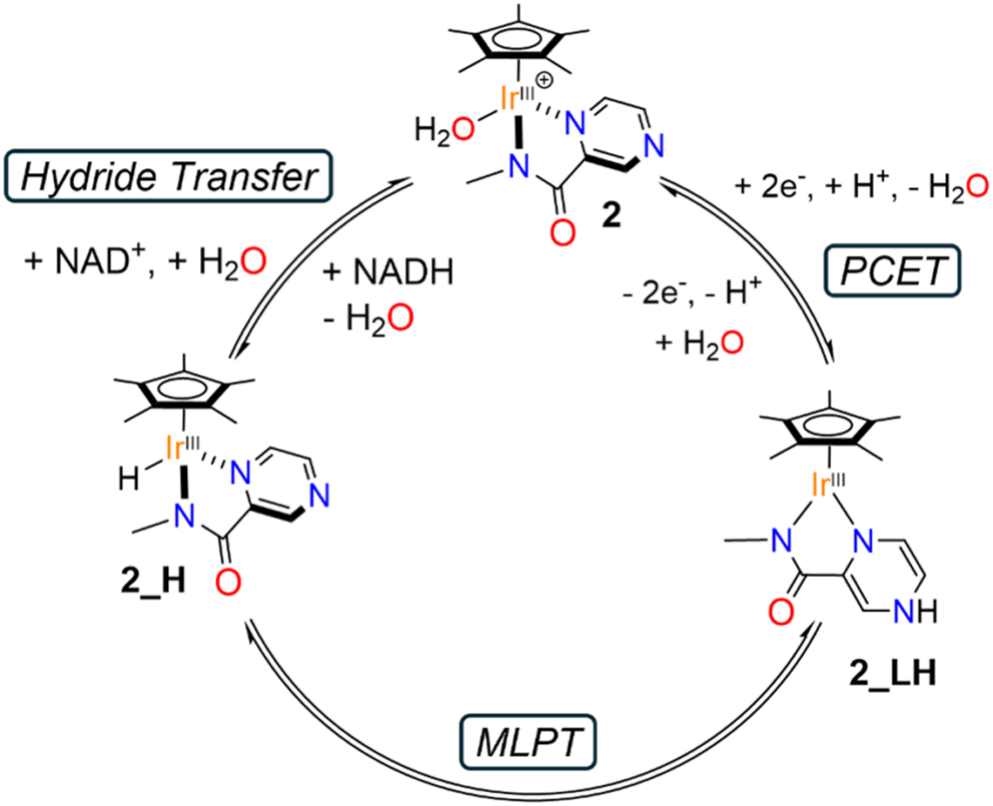
Reaction mechanism proposed for the reversible electrocatalytic NADH/NAD^+^ interconversion mediated by **2**, showcasing the role played by MLPT between **2_LH** and **2_H**.

## Conclusions

This work presents a comprehensive mechanistic investigation of the reversible electrocatalytic interconversion between NAD^+^ and NADH mediated by the iridium complex [Cp*Ir­(N–Me–pyza)­Cl] (**2**). Through a combination of detailed electrochemical and spectroscopic studies, we identified and characterized the key intermediates and individual steps involved in the catalytic cycle. The key finding is that the redox process is centered on the pyrazine ligand, rather than on the metal center, leading to a two–electron, one–proton reduced and dearomatized species (**2_LH**). This intermediate subsequently undergoes a unique type of MLPT to generate the hydride complex (**2_H**). The hydride transfer between **2_H** and NAD^+^ closes the cycle regenerating the starting species.

Dynamic NMR studies and temperature-dependent kinetic measurements enabled quantification of both the equilibrium constant and the kinetic parameters governing the metal–ligand proton tautomerism. Activation parameters extracted from Eyring analysis indicate that the tautomerization proceeds through a highly ordered transition state, likely stabilized by hydrogen bonding with buffer components or cosolutes. The hydride transfer step between **2_H** and NAD^+^ is remarkably rapid and thermodynamically nearly neutral (Δ*G*°_(298)_ = 0.7 ± 0.2 kcal mol^–1^), consistent with highly efficient and reversible catalysis.

Overall, this work introduces a rare and mechanistically distinct example of ligand-centered electrocatalysis, which is pivotal for triggering NAD^+^/NADH interconversion under reversible conditions. The occurrence of a nonclassical MLPT process, in which the redox activity is confined to the ligand scaffold while the oxidation state of the metal remains unchanged, expands the conceptual framework of MLPT and, more importantly, offers a novel design principle for developing electrocatalysts capable of performing multielectron redox transformations with enhanced directionality, tunability, and reversibility.

## Supplementary Material



## Notes

Synthesis, characterization, electrochemical and NMR experiments (PDF)
