# Supplementary material for: Unique Metal–Ligand Proton Tautomerism Underlying the Reversible Electrocatalytic NAD+/NADH Interconversion
Source: J Am Chem Soc. 2026 May 4;148(18):18962–73. doi: 10.1021/jacs.6c00789 (PMC13185114; doi:10.1021/jacs.6c00789)
Supplement: Supplementary file 1 [file ja6c00789_si_001.pdf]

Supporting Information

Unique Metal–Ligand Proton Tautomerism Underlying the Reversible Electrocatalytic NAD$^+$/NADH Interconversion

Gabriel Menendez Rodriguez,[a,] Leonardo Tensi,[b] Elisa Boccalon,[a] Cristiano Zuccaccia,[a] Filippo De Angelis,[c] Luca Rocchigiani,[a] and Alceo Macchioni[a,*]*

[a]*Department of Chemistry, Biology and Biotechnology and CIRCC, University of Perugia, Via Elce di Sotto 8, 06123-Perugia, Italy*

[b]*Department of Pharmaceutical Sciences, University of Perugia, Via del Liceo 1, 06123, Perugia, Italy*

[c]*Department of Chemistry, Biology and Biotechnology and INSTM, University of Perugia, Via Elce di Sotto 8, 06123-Perugia, Italy*

E-mail: gabriel.menendezrodriguez@unipg.it alceo.macchioni@unipg.it

Table of Contents

1. Experimental section ... 3

1.1 General information ... 3

1.2 Synthesis and characterization of 2 ... 3

1.3 *In situ* NMR characterization of 2_H and 2_LH ... 3

1.4 Rate constant determination by EXSY NMR ... 4

1.5 Cyclic Voltammetry ... 8

1.6 Determination of hydride transfer equilibrium constant (K_{HT}) and hydricity ($\Delta G°_{H^-}$) ... 8

1.7 UV-Vis kinetic experiments ... 10

1.8 Open Circuit Potentiometry (OCP) ... 10

2 Figures and tables ... 13

References ... 18

1. Experimental section

1.1 General information

All solvents and reagents were purchased from SIGMA-ALDRICH and used without any further purification. Distilled water was further purified using a Milli-Q Ultrapure water purification system. Britton-Robinson buffers (BRB) were prepared by mixing appropriate volumes of stock solutions of HCOOH (40 mM), H_3BO_3 (40 mM) and $Na_{3-x}H_xPO_4$ (x = 1 or 2, 40 mM). Phosphate buffer (0.1 M, pH 7) was prepared by mixing appropriate volumes of previously prepared acid and base stock solutions (0.1 M). Tris-(hydroxymethyl)aminomethane (Tris)/HCl buffer (0.1 M, pH 7) was prepared by adding concentrated HCl to a stock solution of Tris (0.1 M). $[Cp^*IrCl_2]_2$ was prepared according to the literature.[1] NMR spectra were recorded on a Bruker Avance III 400 spectrometer equipped with a smartprobe (400 MHz for ^1H) with a z gradient coil or on a Bruker Avance NEO 600 spectrometer equipped with the Prodigy™ Bruker Cryoprobe (600 MHz for ^1H) with a z gradient coil. Residual solvent resonances were used for referencing; reported chemical shifts are relative to external TMS (for ^1H and ^{13}C) and NH_3 (for ^{15}N).

X-Ray diffraction pattern of **2** was recorded using a Bruker D8 Venture diffractometer equipped with an Incoatec ImuS3.0 microfocus sealed-tube MoKα (λ = 0.71073 Å) source and a CCD Photon II detector. The data collected through generic φ and ω scans were integrated and reduced using the Bruker AXS V8 Saint Software. The structure was solved, and all the thermal parameters were anisotropically refined using the SHELXT and SHELXL packages of the Bruker APEX3 software.

1.2 Synthesis and characterization of 2

80 mg of $[Cp^*IrCl_2]_2$ (0.1 mmol), 27.6 mg of N-methylpyrazine-2-carboxamide (2.0 eq.) and 11.3 mg of KOH were suspended in 10 mL of methanol. The reaction mixture was stirred at room temperature for 2 hours; the solvent was then removed under reduced pressure. The collected solid was contacted with 5 ml of CH_2Cl_2 to extract the product. The CH_2Cl_2 solution was filtered and reduced in volume through a gentle flux of nitrogen. Single crystals of **2** suitable for X-Ray diffraction studies (Table S1) were obtained by slow diffusion of diethyl ether into the saturated solution of the complex in dichloromethane. Yield = 81 mg (81%). X-ray Crystal Structure: CCDC 2467679 data can be obtained free of charge from the Cambridge Crystallographic Data Centre.

^1H NMR (400 MHz, CD_2Cl_2, 298 K, δ in ppm, J in Hz): δ = 9.17 (d, $^5J_{HH}$ = 1.3, H3), 8.71 (d, $^3J_{HH}$ = 3.2, H5), 8.53 (dd, $^3J_{HH}$ = 3.2, $^5J_{HH}$ = 1.3, H6), 3.32 (s, H8), 1.73 (s, H1). $^{13}C\{^1H\}$ NMR (100 MHz, CD_2Cl_2, 298 K, δ in ppm): δ = 168.8 (s, C7), 148.7 (s, C2), 147.9 (s, C3), 147.6 (s, C5), 142.4 (s, C6), 87.5 (s, C9), 36.3 (s, C8), 8.7 (s, C10). $^{15}N\{^1H\}$ NMR (40 MHz, CD_3OD, 298 K, δ in ppm): δ = 337.7 (s, N4), 244.9 (s, N1), 125.4 (s, NMe).

1.3 *In situ* NMR characterization of 2_H and 2_LH

Complex **2** (5.0 mg, 0.01 mmol) and HCOOK (16.8 mg, 0.2 mmol) were placed in a J-Young NMR tube, followed by the addition of deoxygenated CD_3OD (0.6 mL). The reaction of **2** with formate affords a clean **2_H/2_LH** mixture, which remains stable for weeks, enabling full NMR characterization. The Ir-H resonance of **2_H** was identified by repeating the reaction in CH_3OH instead of CD_3OD. An additional experiment was carried out in aprotic DMSO-d6 to demonstrate the presence of the H4 resonance (Figure S5d).

Data for **2_H**: ^1H NMR (400 MHz, CD_3OD, 298 K, δ in ppm, J in Hz): δ = 8.87 (d, $^5J_{HH}$ = 1.2, H3), 8.85 (dd, $^5J_{HH}$ = 1.2, $^3J_{HH}$ = 3.3, H6), 8.44 (d, $^3J_{HH}$ = 3.2, H5), 3.33 (s, H8), 1.92 (s, H1). $^{13}C\{^1H\}$ NMR (100 MHz, CD_3OD, 298 K, δ in ppm): δ = 168.9 (s, C7), 148.9 (s, C2), 146.3 (s, C5), 146.2 (s, C3),

145.3 (s, C6), 89.7 (s, C9), 38.6 (s, C8), 8.4 (s, C10). $^{15}N\{^1H\}$ NMR (40 MHz, CD_3OD, 298 K, δ in ppm): δ = 324.8 (s, N4), 237.2 (s, N1), 112.6 (s, NMe).

Data for **2_LH**: ^1H NMR (400 MHz, CD_3OD, 298 K, δ in ppm, J in Hz): δ = 7.16 (d, $^3J_{HH}$ = 5.9, H6), 6.89 (d, $^4J_{HH}$ = 1.6, H3), 5.49 (dd, $^3J_{HH}$ = 5.9, $^4J_{HH}$ = 1.6, H5), 3.57 (s, H8), 1.96 (s, H1). $^{13}C\{^1H\}$ NMR (100 MHz, CD_3OD, 298 K, δ in ppm): δ = 172.6 (s, C7), 136.6 (s, C6), 131.4 (s, C2), 127.1 (s, C3), 114.5 (s, C5), 83.6 (s, C9), 39.2 (s, C8), 8.5 (s, C10). $^{15}N\{^1H\}$ NMR (40 MHz, CD_3OD, 298 K, δ in ppm): δ = 171.4 (s, N1), 151.0 (s, NMe), 105.5 (s, N4).

1.4 Rate constant determination by EXSY NMR

Metal-Ligand Proton Tautomerism

To determine the kinetic constants of the **2_LH⇄2_H** equilibrium, one-dimensional (1D) EXSY NMR measurements on a solution of **2** (4 mM) in 0.1 M PBS (pH 7, H_2O/D_2O 90:10) or 0.1 M Tris/HCl (pH 7, H_2O/D_2O 90:10) and ca. 20 equivalents of HCOOK were acquired by using a modified version of the "selnogp" pulse program (from Bruker library) in which a presaturation pulse during the relaxation delay was added. All experiments were carried out with a recycle delay of 5 s and all the FIDs were acquired using 32k points, 16 scans and were processed with a line broadening of 3.0 Hz with zero-filling to 64k points.

For each temperature four experiments were carried out. First, two reference spectra were recorded with a mixing time (τ) of 0 s, selectively exciting the H6 proton of **2_H** (Figure S1b) and **2_LH** (Figure S1c), in order to determine the initial signal intensities in the absence of magnetization transfer (I_A^0 and I_B^0 respectively).

Subsequently, two additional spectra were acquired, one selectively exciting the H6 proton of **2_H** (Figure S1d) and the other the H6 proton of **2_LH** (Figure S1e), using a mixing time sufficiently long to allow magnetization exchange between the sites involved in the **2_LH⇄2_H** equilibrium. The mixing times employed were 0.8 s, 0.6 s, 0.4 s, 0.3 s, and 0.2 s at 278 K, 283 K, 288 K, 293 K, and 298 K, respectively.

These experiments, recorded at longer τ values, enabled observation of the exchange cross-peaks (I_{AB} and I_{BA}) together with the corresponding decrease in the diagonal peak intensities (I_{AA} and I_{BB}).

The selected signals were integrated relative to I_A^0 (set to 100), and the magnetization transfer rate constants k^1 and k^{-1} were determined using the EXSYCALC software.[2]

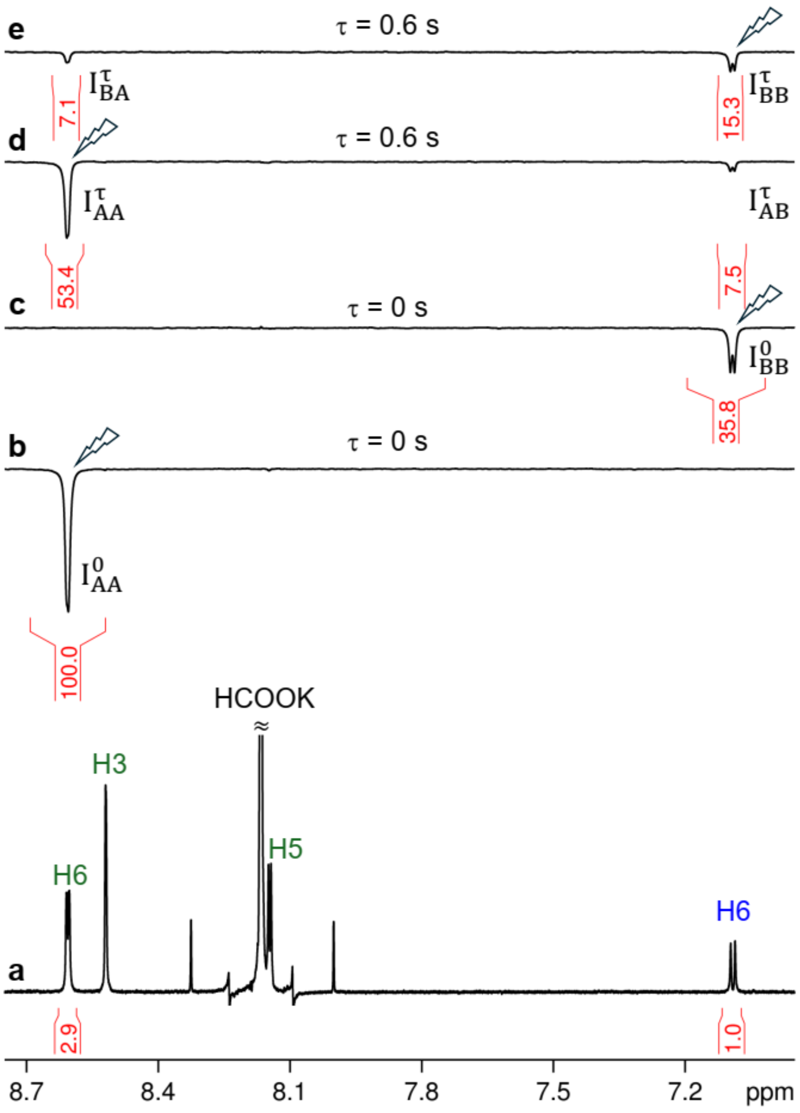

Figure S1. a) A section of the ^{1}H NMR spectrum showing the resonances of **2_H** (H6 green) and **2_LH** (H6 blue) used to determine the kinetic constants. b) 1D EXSY NMR spectrum recorded at $\tau = 0$, selectively exiting H6 of **2_H**. c) 1D EXSY NMR spectrum recorded at $\tau = 0$, selectively exiting H6 of **2_LH**. d) 1D EXSY NMR spectrum recorded at $\tau = 0.6$ s, selectively exiting H6 of **2_H**. e) 1D EXSY NMR spectrum recorded at $\tau = 0.6$ s, selectively exiting H6 of **2_LH**. Conditions: 0.1 M PBS (pH 7), H_2O/D_2O 90:10, 283 K.

The following procedure was used for the determination of rate constant errors:

1) Two independents EXSY NMR measurements (Sample 1-2, Table S1) were performed at 283 K on a deoxygenated solution of **2** (4 mM) and HCOOK (80 mM) in phosphate buffer (0.1 M, pH 7, H_2O/D_2O 90:10) in order to determine k_1 and k_{-1} This temperature was selected because it represents the most critical case in terms of the differences observed relative to the values reported at 278 K and 288 K (Table 1 in the main manuscript).
2) Each spectrum was phase corrected manually and baseline corrections were applied using the instrument software.
3) Relative peak integrations in the 1D EXSY NMR spectra were performed manually.
4) Steps 2) and 3) were repeated four times for each sample, yielding the values reported in Table S1.

5) The eight resulting values of k_1 and k_{-1} were averaged ($\bar{k}$), and the corresponding standard deviations (σ) were calculated.
6) The relative error at a 95% confidence level (RE_{95}) was calculated as $2\sigma/\bar{k}$ and applied to the rate constant values reported in Table 1 of the manuscript.

Table S1. Data used to determine the kinetic constants of MLPT and their associated error.

	Entry	τ (s)	I_{AA}	I_{AB}	I_{BB}	I_{BA}	k_1 (s^{-1})	k_{-1} (s^{-1})
Sample 1	1	0	100		35.8		0.746	0.253
		0.6	53.4	7.5	15.3	7.1		
	2	0	100		36.3		0.719	0.240
		0.6	52.9	7.4	15.9	6.8		
	3	0	100		34.5		0.823	0.236
		0.6	54.8	8.2	15.2	6.8		
	4	0	100		36.3		0.717	0.243
		0.6	53.9	7.6	16.6	7.1		
Sample 2	5	0	100		34.8		0.761	0.245
		0.6	54.8	8	16.8	7.4		
	6	0	100		34.9		0.754	0.253
		0.6	54.5	7.8	16.3	7.5		
	7	0	100		35.6		0.759	0.267
		0.6	54.9	7.8	15.7	7.7		
	8	0	100		35.5		0.720	0.256
		0.6	53.5	7.4	16.1	7.4		
						$\bar{k}$	0.750	0.249
						σ	0.035	0.010
						RE_{95}	0.093	0.081

Hydride transfer reaction

The rate constants for the reaction **2_H + NAD⁺⇄2 + NADH** were determined following an analogous procedure to that reported above for the MLPT process.

In this case, a solution containing NAD^+ (3.0 mM), NADH (3.0 mM) and **2** (0.5 mM) in BRB (0.04 M, pH 7) was prepared, and the magnetization transfer rates between NAD^+ and NADH (k_{M2} and k_{M-2}) were measured by monitoring the chemical exchange between H2 proton of NAD^+ (9.23 ppm) and the corresponding proton of 1,4-NADH (6.85 ppm; H2), as shown in Figure S2. In this case, experiments were performed at 298 K using a mixing time of 0.8 s. The selected signals were integrated relative to I_A^0 (Figure S2), and the magnetization transfer rate constants k_{M2} and k_{M-2} were determined using the EXSYCALC software[2], yielding values of 0.43 s^{-1} and 0.51 s^{-1}, respectively.

The second order rate constant of the forward (k_2) and backward (k_{-2}) reactions were then derived by dividing the forward and backward magnetization transfer rates by the concentrations of **2_H** and **2**, respectively (Eq. S1-S2).

$k_2 =$ [illegible] S1

$k_2 = $ [illegible]

S2

Where the concentrations of [**2_H**] and [**2**] (0.30 mM and 0.12 mM, respectively) were calculated from the experimental concentrations of NAD^+ and NADH (1,4-NADH + 1,6-NADH; Figure S2a) and from the independently determined equilibrium constants K_{HT} and K_{MLPT}. A relative error of 0.14 was calculated based on the uncertainty associated with the integration of both the ^1H NMR (Figure S2a) and 1D EXSY spectra (Figure S2b-e).

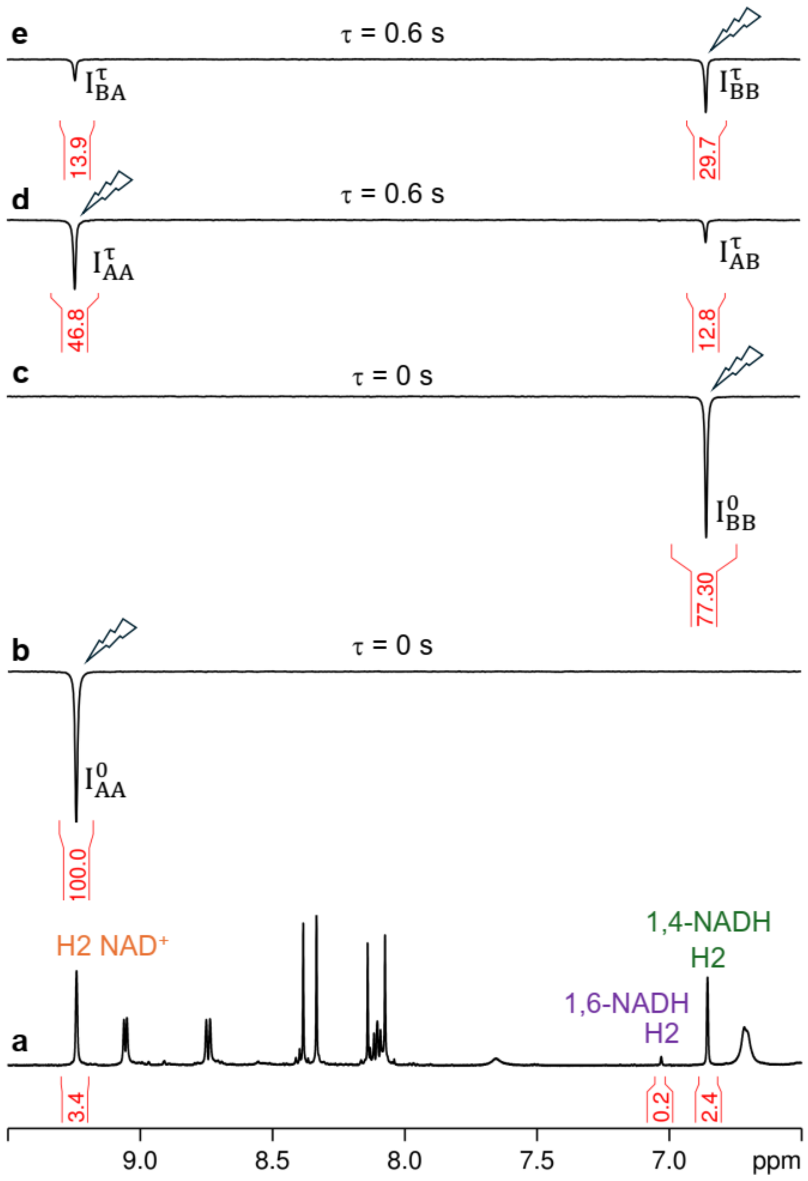

Figure S2. a) A section of the ^1H NMR spectrum showing the resonances of NAD^+ (H2 orange) and NADH (H2 green) used to determine the kinetic constants and their relative concentrations (mM). b) 1D EXSY NMR spectrum recorded at τ = 0, selectively exiting H2 of NAD^+. c) 1D EXSY NMR spectrum recorded at τ = 0, selectively exiting H2 of NADH. d) 1D EXSY NMR spectrum recorded at τ = 0.8 s, selectively exiting H2 of NAD^+. e) 1D EXSY NMR spectrum recorded at τ = 0.8 s, selectively exiting H2 of NADH. Conditions: 0.04 M BRB (pH 7), H_2O/D_2O 90:10, 298 K.

1.5 Cyclic Voltammetry

Cyclic voltammetry (CV) experiments were carried out under an atmosphere of nitrogen using a Squidstat Plus potentiostat (manufactured by Admiral Instruments). In all experiments, a water jacketed three-electrode electrochemical cell with a capacity of 20.0 mL equipped with a $Hg/HgSO_4$ (Sat'd K_2SO_4) reference electrode, a platinum wire as the counter electrode and a 3 mm glassy carbon working electrode (CH Instruments) were used. No iR-correction was applied. Potentials in aqueous solutions were referenced to the normal hydrogen electrode (NHE) using the formula [illegible] where [illegible] = +0.65 V. Glassy carbon working electrode was mechanically polished with 0.05 micron alumina for 15-20 seconds, thoroughly rinsed with ultrapure water and gently polished with a Kimwipe to remove water from the surface.

1.6 Determination of hydride transfer equilibrium constant (*K_{HT}*) and hydricity (*$\Delta G°_{H^-}$*)

In order to determine the hydride transfer equilibrium constant (K_{HT}, Eq. S3) and hydricity of **2_H** ($\Delta G°_{H^-}$), NMR samples containing initial concentration of **2** (3.0 mM) and 1,4-NADH (3.0 mM) in deaerated Britton-Robinson buffer (40.0 mM, pH 7, H_2O/D_2O 90:10, 298 K) or Tris/HCl buffer (0.1 M, pH 7, H_2O/D_2O 90:10, 298 K) were prepared and analyzed by ^1H NMR spectroscopy. [NADH] and [NAD^+] were directly determined by relative integration of the diagnostic resonances of the nicotinamide ring of 1,4-NADH (6.85 ppm; H2) and NAD^+ (9.23 ppm, H2), as shown in Figure S3.

K_{HT} = [illegible] (S3)

[**2**] and [**2_H**] were calculated based on the reaction stoichiometry and the speciation arising from the tautomeric equilibrium using Eq. S4-S6.

[illegible] (S4)

[illegible] (S5)

[illegible] (S6)

Eq. S4-S6 were then introduced into Eq. S3, finally leading to Eq. S7.

K_{HT} = [illegible] (S7)

The relative uncertainty of K_{HT} ([illegible]), was determined using Equation S8:

[illegible] (S8)

where [illegible] and [illegible] represent the relative uncertainties in the concentrations of NADH and NAD^+, respectively. These uncertainties arise from the integration of the ^1H NMR signals and were estimated to be 0.1 for both species.

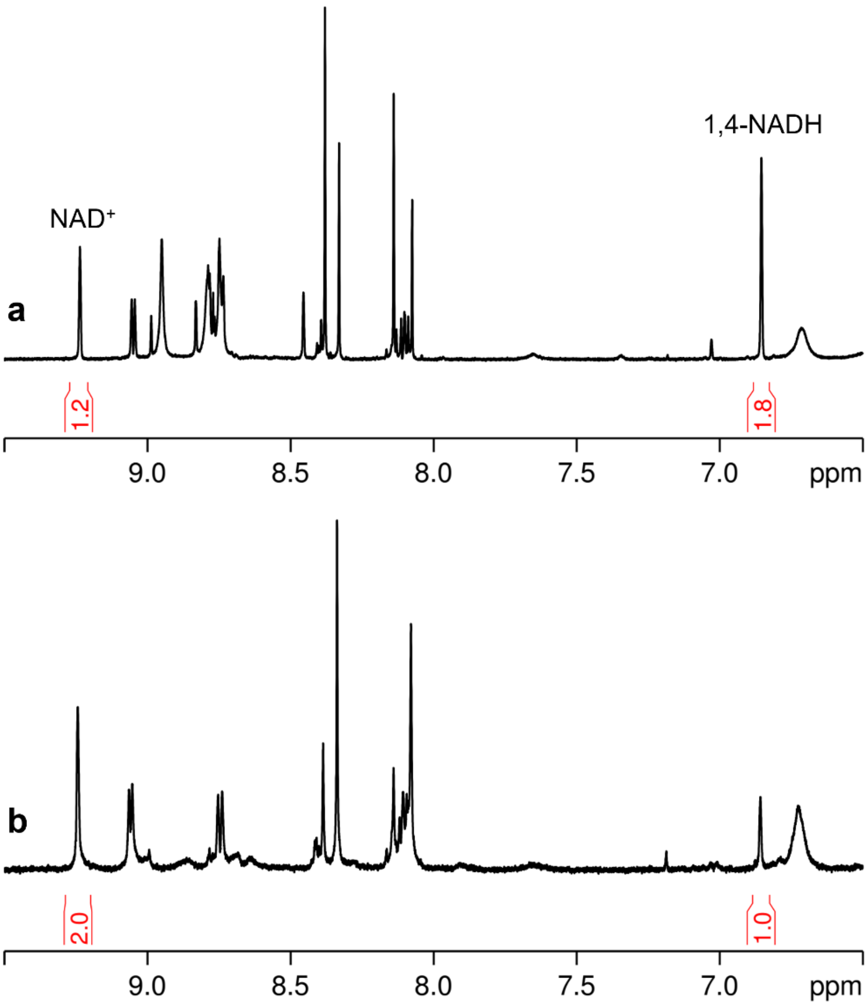

Figure S3. ^{1}H NMR spectra of the solution of **2** (3 mM) with NADH (3 mM) in a) Tris/HCl buffer (0.1 M, pH 7, H_2O/D_2O 90:10, 298 K) or in b) Britton-Robinson buffer (40.0 mM, pH 7, H_2O/D_2O 90:10, 298 K).

The hydricity of the **2_H** was then determined by using the thermochemical cycle shown in Scheme S1, where $\Delta G°_{rxn}$ = -1.364 log(K_{HT}) and $\Delta G°_{NADH}$ = 28.9 kcal/mol.[3] The uncertainty in the hydricity value was determined by error propagation associated with NMR integrations and concentration determinations.

The ligand replacing the hydride at the Ir center was not explicitly represented in Scheme S1, as its identity is expected to arise from multiple equilibria involving not only solvent molecules but also components of the BRB buffer system (acetate and/or phosphate anions), or Cl^- in the case of Tris/HCl buffer.

Scheme S1. Thermochemical cycle for determining $\Delta G°_{H^-}$ (kcal/mol).

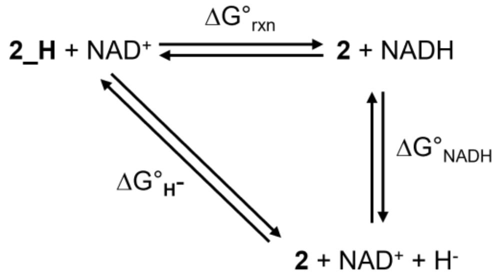

1.7 UV-Vis kinetic experiments

A deoxygenated solution of **2** (0.1 mM, 2.5 mL) in BRB (40 mM, pH 7) was transferred into a quartz cuvette, and the system was allowed to equilibrate under stirring for 20 min at 25 °C. After background correction, 0.5 mL of a solution of NADH (10 mM) in BRB (40 mM, pH 7) was injected (final volume = 3 mL) and the evolution of the spectrum was followed at λ = 450 nm. The experiment was repeated twice.

1.8 Open Circuit Potentiometry (OCP)

Open circuit potentiometry (CPE) experiments were performed using the same setup as for CV experiments. In a typical experiment, the electrochemical cell was filled with a solution of **2** (0.5 mM, 5 mL) in BRB buffer (0.04 M, pH 7), which was purged with nitrogen to remove dissolved oxygen and allowed to equilibrate under continuous stirring (5000 rpm). Stirring was then stopped, and a cyclic voltammogram was recorded at 5 mV s^{-1} to determine the experimental value of $E_{1/2}$(Figure S9). Subsequently, stirring was resumed and the open circuit potential (OCP) was monitored for at least 120 seconds before 0.5 – 4 eq. of NADH was added, injecting different volumes of a freshly prepared solution of NADH (10 mM) in BRB (40 mM, pH 7). OCP values were taken 100 seconds after each addition to ensure that equilibrium was reached. Experiments were performed at least in duplicate.

To construct the theoretical curves shown in Figure 7a, we first solved the system of five independent equations (S9) and five unknown variables ([**2**], [**2_H**], [**2_LH**], [NADH] and [NAD^+]) for different values of initial NADH concentration ([NADH]°), while keeping the initial concentration of **2** ([2]° = 0.5 mM) constant. The equilibrium constants K_{HT} and K_{MLPT} were fixed at 0.3 and 4.0, respectively, based on NMR results.

[illegible] (S9)

This system was solved using the substitution method until isolation of [**2_LH**] was achieved (Eq. S10).

[illegible] (S10)

Second order equation S10 was solved using the quadratic formula:

[illegible] (S11)

Where:

$a = $ [illegible] (S12)

$b = $ [illegible] (S13)

$c = $ [illegible] (S14)

After finding the value of [**2_LH**], back-substitution into the equations S9 allowed the determination of the remaining variables.

The resulting values of [**2**], [**2_H**] and [**2_LH**] were then used in the Nernst equation 8 (for scenario A) and equation 9 (for scenario B) of the main manuscript. Table S2 provides the solutions of the system of equations S9 for different equivalents of NADH with respect to the initial concentration of **2**, as well as the values of OCP-$E_{1/2}$ (mV) used to construct the theoretical curves shown in Figure 7.

Table S2. Analytical solutions of the system of equations S9 for different equivalents of NADH with respect to the initial concentration of **2** (0.5 mM).

eq. NADH	[2_LH]	[2_H]	[2]	[NADH]	[NAD^+]	Scenario A OCP-$E_{1/2}$ (mV)	Scenario B OCP-$E_{1/2}$ (mV)
0.20	0.019	0.076	0.405	0.005	0.095	21.5	39.3
0.30	0.027	0.110	0.363	0.013	0.137	15.3	33.1
0.40	0.035	0.141	0.323	0.023	0.177	10.6	28.4
0.50	0.042	0.170	0.288	0.038	0.212	6.8	24.6
0.60	0.049	0.195	0.256	0.056	0.244	3.5	21.3
0.70	0.054	0.218	0.228	0.078	0.272	0.6	18.4
0.80	0.059	0.237	0.204	0.104	0.296	-2.0	15.8
0.90	0.063	0.254	0.183	0.133	0.317	-4.2	13.6
1.00	0.067	0.268	0.164	0.164	0.336	-6.3	11.5
1.10	0.070	0.281	0.149	0.199	0.351	-8.2	9.6
1.20	0.073	0.292	0.135	0.235	0.365	-9.8	8.0
1.30	0.075	0.301	0.124	0.274	0.376	-11.4	6.4
1.40	0.077	0.309	0.114	0.314	0.386	-12.8	5.0
1.50	0.079	0.316	0.105	0.355	0.395	-14.1	3.7
1.60	0.080	0.322	0.098	0.398	0.402	-15.3	2.5
1.70	0.082	0.327	0.091	0.441	0.409	-16.4	1.4
1.80	0.083	0.332	0.085	0.485	0.415	-17.5	0.3
1.90	0.084	0.336	0.080	0.530	0.420	-18.4	-0.6
2.00	0.085	0.340	0.075	0.575	0.425	-19.4	-1.6
2.10	0.086	0.343	0.071	0.621	0.429	-20.2	-2.4
2.20	0.087	0.346	0.067	0.667	0.433	-21.0	-3.2
2.30	0.087	0.349	0.064	0.714	0.436	-21.8	-4.0
2.40	0.088	0.351	0.061	0.761	0.439	-22.5	-4.7
2.50	0.088	0.354	0.058	0.808	0.442	-23.2	-5.4
2.60	0.089	0.356	0.055	0.855	0.445	-23.9	-6.1
2.70	0.089	0.358	0.053	0.903	0.447	-24.5	-6.7
2.80	0.090	0.359	0.051	0.951	0.449	-25.1	-7.3
2.90	0.090	0.361	0.049	0.999	0.451	-25.7	-7.9
3.00	0.091	0.362	0.047	1.047	0.453	-26.2	-8.4
3.10	0.091	0.364	0.045	1.095	0.455	-26.7	-8.9
3.20	0.091	0.365	0.044	1.144	0.456	-27.3	-9.5
3.30	0.092	0.366	0.042	1.192	0.458	-27.7	-9.9
3.40	0.092	0.367	0.041	1.241	0.459	-28.2	-10.4
3.50	0.092	0.368	0.039	1.289	0.461	-28.7	-10.9
3.60	0.092	0.369	0.038	1.338	0.462	-29.1	-11.3
3.70	0.093	0.370	0.037	1.387	0.463	-29.5	-11.7
3.80	0.093	0.371	0.036	1.436	0.464	-30.0	-12.2
3.90	0.093	0.372	0.035	1.485	0.465	-30.4	-12.6
4.00	0.093	0.373	0.034	1.534	0.466	-30.8	-13.0

2 Figures and tables

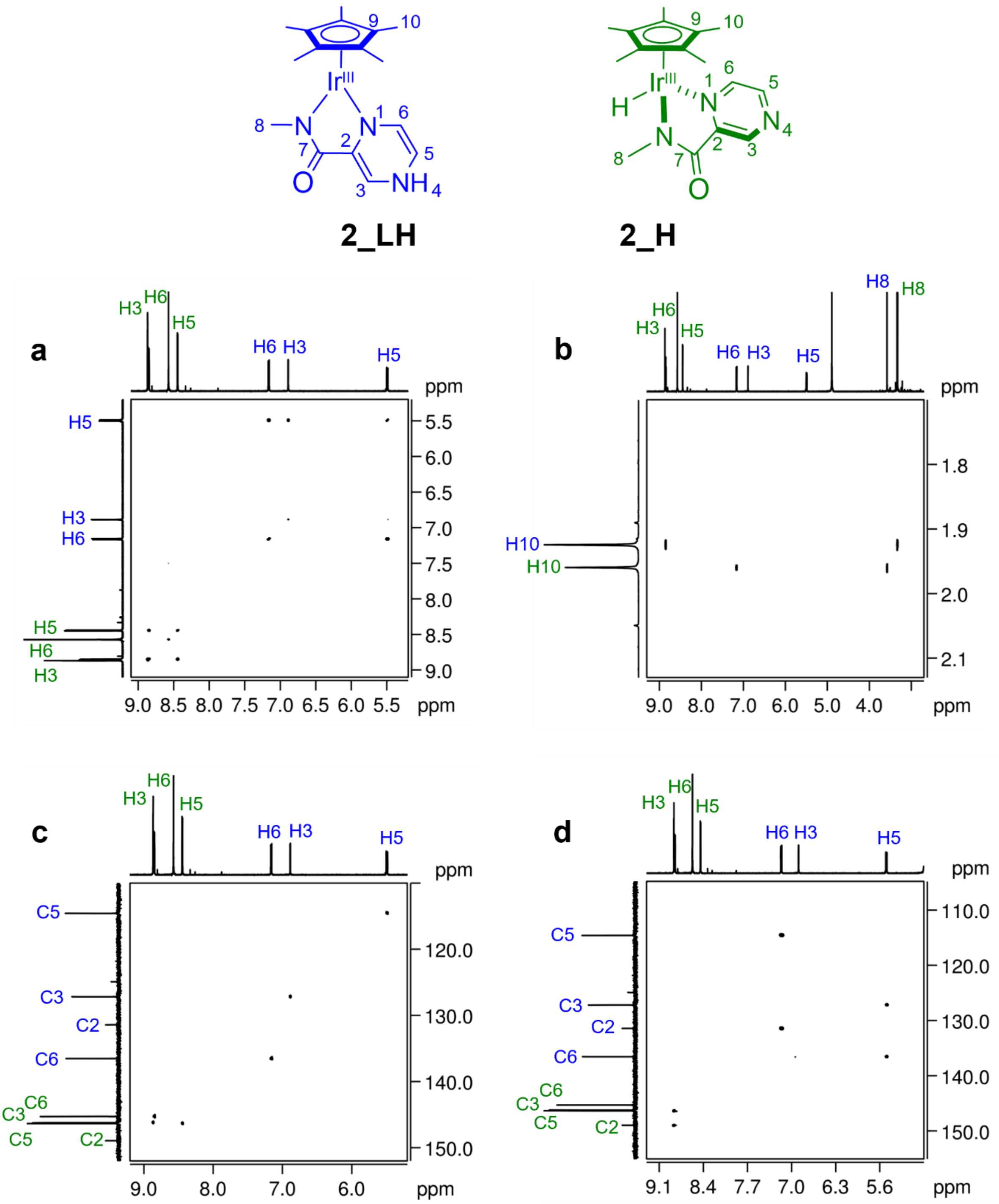

Figure S4. ^{1}H COSY (a), ^{1}H NOESY (b), ^{1}H-^{13}C HSQC (c) and ^{1}H-^{13}C HMBC (d) NMR spectra of the **2_H**/**2_LH** mixture (CD_3OD, 298 K).

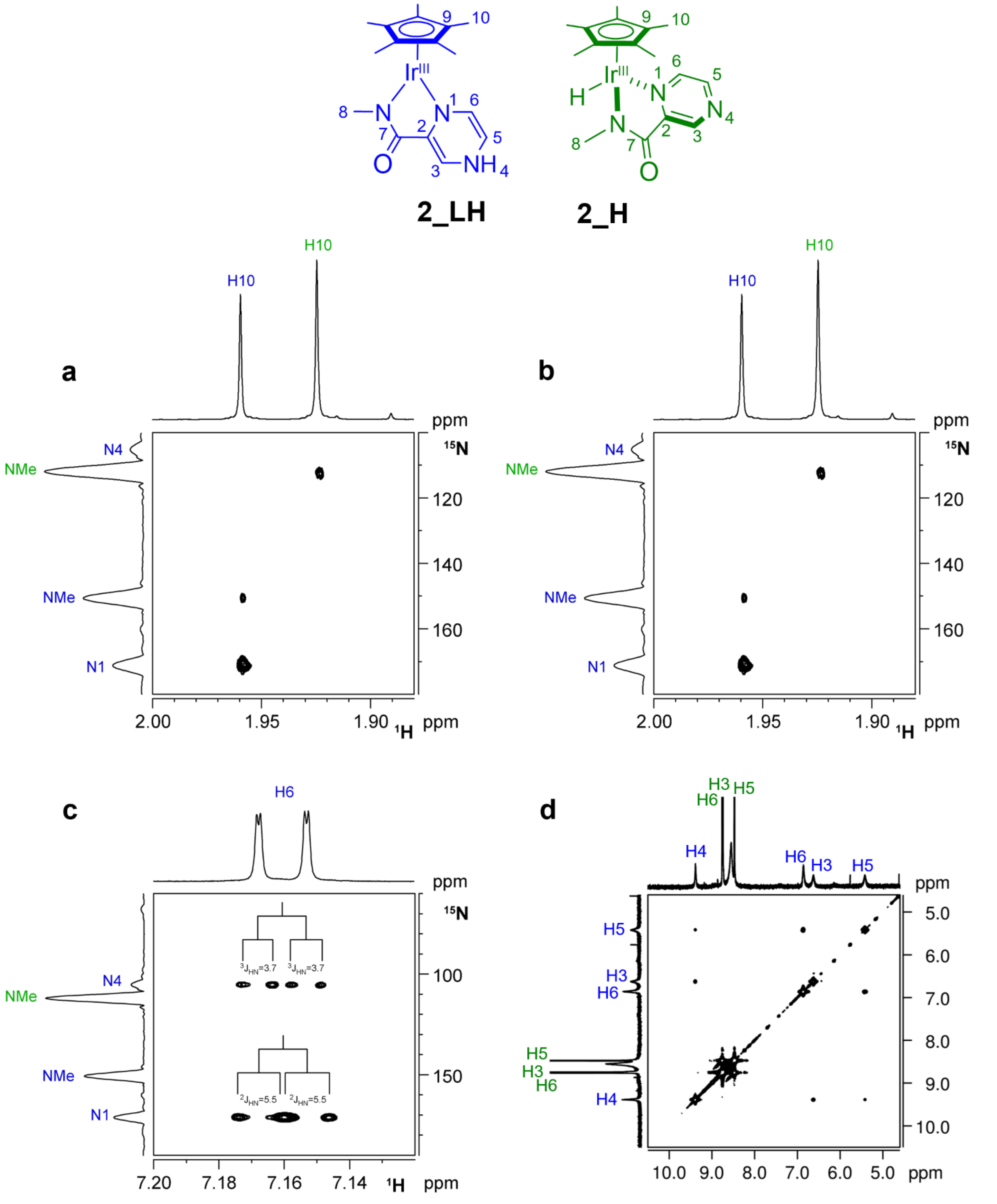

Figure S5. (a-c) ^{1}H-^{15}N HMBC experiments used for the assignment nitrogen atoms of **2_H** and **2_LH** (CD_3OD, 298 K). d) ^{1}H COSY experiment showing the scalar correlation between H4 and H3 and H5 in **2_LH** (DMSO-*d6*, 298K).

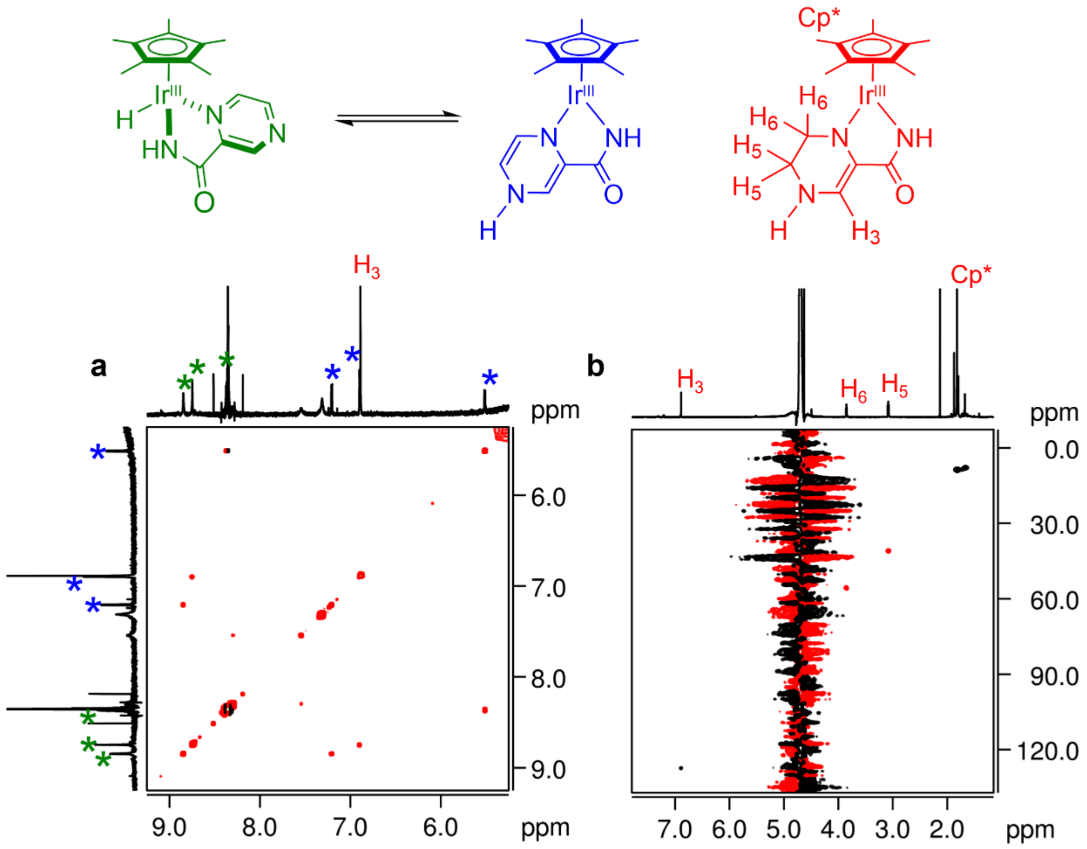

Figure S6. a) ^{1}H EXSY spectrum showing the dynamic equilibrium between **1_H** and **1_LH**. b) ^{1}H-^{13}C HSQC spectrum showing ^{1}H/^{13}C scalar correlations in the over-reduced pyrazine ligand.

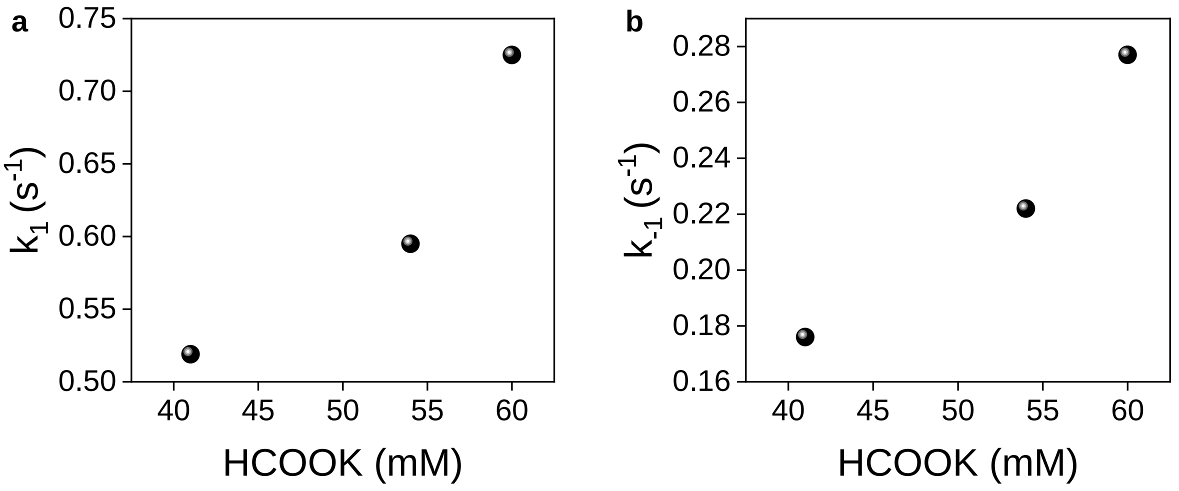

Figure S7. Effect of HCOOK concentration on the a) forward and b) backward kinetic constants of the MLPT.

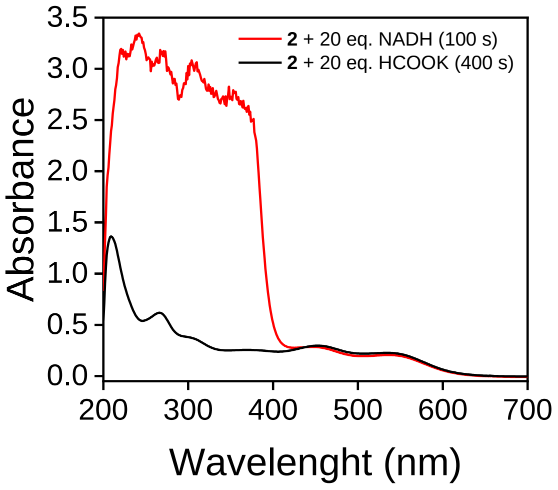

Figure S8. UV-vis spectra obtained after the reaction of **2** with 20 eq. of NADH (red) and HCOOK (black).

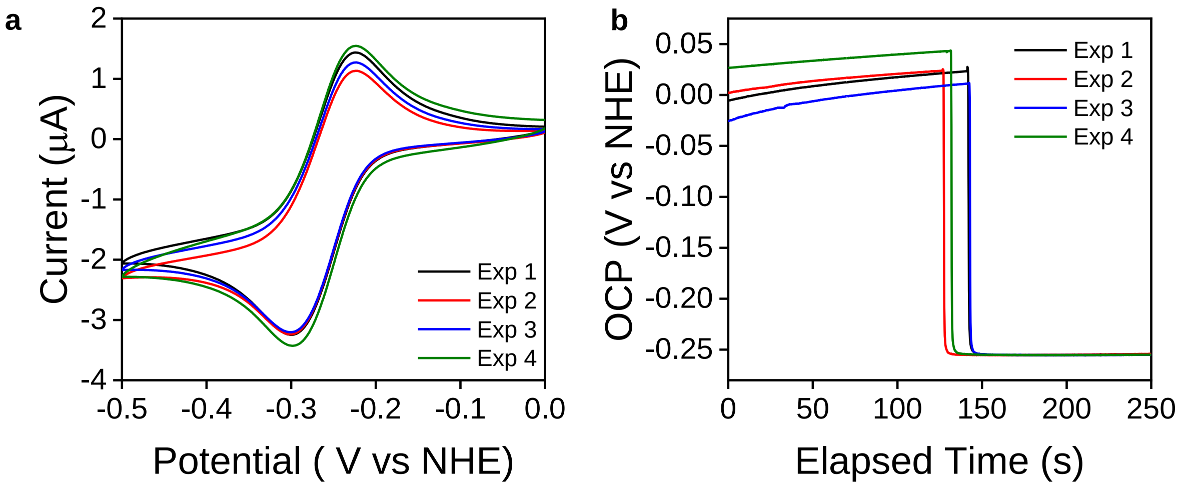

Figure S9. a) CVs recorded at 5 mV/s to determine $E_{1/2}$ before each OCP experiment. Conditions: [**2**] = 0.5 mM, BRB (0.04 M, pH 7), N_2 atmosphere, 298 K. b) Replicates of OCP experiments for the reaction of **2** (0.5 mM) in BRB (0.04 M, pH 7) with 1 eq of NADH.

Table S3. Crystal data and structure refinement for **2**.

**Complex**	**[Cp*Ir(NMe-pyza)Cl] (2)**
**CCDC n.**	[illegible]
Elemental Formula	[illegible]
Formula weight	517.02
Crystal system	Monoclinic
Space group	P2(1)/c
Unit cell dimensions:	
a = (Å)	[illegible]
b =	[illegible]
c =	[illegible]
β = (°)	[illegible]
Volume (Å^3)	[illegible]
Z, Calculated density (g/cm^3)	[illegible]
F(000)	[illegible]
Absorption coefficient (mm^{-1})	[illegible]
Temperature (K)	[illegible]
Crystal colour, shape	[illegible]
Crystal size (mm)	[illegible]
On the diffractometer:	
Theta range for data collection	[illegible]
Limiting indices	[illegible]
Completeness	[illegible]
Max. and min. transmission	[illegible]
Reflns collected (not incl. absences)	[illegible]
No. of unique reflns, R(int) for equivs	[illegible]
No. of 'observed' reflns (I > 2σI)	[illegible]
Refinement:	
Data/restraints/parameters	[illegible]
Goodness-of-fit on F^2	[illegible]
Final R indices ('obsd' data)	[illegible]
Final R indices (all data)	[illegible]
Reflns weighted: 1/w = [a]	[illegible]
Largest diff. peak and hole (e. Å^{-3})	[illegible]

[a]where P=(Fo^2^+2Fc^2^)/3

References

(1) Ball, R. G.; Graham, W. A. G.; Hoyano, J. K.; McMaster, A. D.; Mattson, B. M.; Heinekey, D. M.; Mattson, B. M.; Michel, S. T. Synthesis and Structure of [(η-C5Me5)Ir(CO)]2. *Inorg. Chem.* **1990**, *29* (10), 2023–2025. https://doi.org/10.1021/IC00335A051/SUPPL_FILE/IC00335A051_SI_001.PDF.

(2) *Freeware Tools for Enhanced Workflows - Mestrelab Research Analytical Chemistry Software Freeware*. https://mestrelab.com/main-product/freeware (accessed 2026-02-18).

(3) Wiedner, E. S.; Chambers, M. B.; Pitman, C. L.; Bullock, R. M.; Miller, A. J. M.; Appel, A. M. Thermodynamic Hydricity of Transition Metal Hydrides. *Chem. Rev.* **2016**, *116* (15), 8655–8692. https://doi.org/10.1021/ACS.CHEMREV.6B00168/ASSET/IMAGES/MEDIUM/CR-2016-00168P_0029.GIF.
